# The response of wheat and its microbiome to contemporary and historical water stress in a field experiment

**DOI:** 10.1038/s43705-022-00151-2

**Published:** 2022-07-27

**Authors:** Hamed Azarbad, Luke D. Bainard, Asmaâ Agoussar, Julien Tremblay, Etienne Yergeau

**Affiliations:** 1 Department of Biology, Evolutionary Ecology of Plants, Philipps-University Marburg, Karl-von-Frisch-Strasse 8, 35043 Marburg, Germany; 2 Agassiz Research and Development Centre, Agriculture and Agri-Food Canada, 6947 #7 Highway, Agassiz, BC Canada; 3 Centre Armand-Frappier Santé Biotechnologie, Institut national de la recherche scientifique, Laval, QC Canada; 4 Energy, Mining and Environment, National Research Council Canada, Montréal, QC Canada

**Keywords:** Microbial ecology, Climate-change ecology

## Abstract

In a field experiment, we evaluated the impact of 37 years of contrasting water stress history on the microbial response in various plant compartments at two distinct developmental stages when four wheat genotypes were exposed to contemporary water stress. Seeds were collected and sampled at the end of the experiment to characterize endophytic and epiphytic microbial communities. Amplicon sequencing data revealed that plant development stage and water stress history were the main factors shaping the microbiome of the major plant parts in response to contemporary water limitation. Our results indicate that seeds can become colonized by divergent microbial communities within a single generation based on the initial pool of microbes as determined by historical contingencies, which was modulated by the contemporary environmental conditions and the plant genotype. Such information is essential to incorporate microbial-based strategies into conventional plant breeding to enhance plant resistance to stress.

## Introduction

One of the effects of climate change is the increased frequency and severity of extreme weather events [1]. Among these, drought has increased substantially in recent years and dramatically reduced crop yields [2]. The decline in the production of major crops stands in strong contrast to the need to increase global food production by up to 70% or more by 2050 to feed a growing human population [3]. Therefore, there is an urgent need to improve crop production under sub-optimal conditions.

One interesting approach to enhance plant resilience and mitigate the impacts of disturbance on yields is through the manipulation of plant-associated microorganisms [4–6]. Indeed, beneficial microbes associated with different plant parts play critical roles in improving nutrient uptake, inhibiting pathogens, and protecting plants against stresses [7]. Beneficial soil- and plant-associated microbes can also modulate the physiological response of plants to water stress and improve plant adaptation. However, before being able to manipulate microbial communities for plant water stress adaptation, we must understand how they respond to water limitations and how this is constrained by initial microbial communities and plant genotype.

There are two main ways by which the plant microbial community is assembled: vertically (from parent plants to offspring) and horizontally (from the environment such as soil, air, water, and insects). Soil- and seed-borne microorganisms are the major contributors to the assembly of the plant microbial communities [8–11]. Previous exposure to water stress of these early colonists could have direct consequences on the plants and on the degree to which plants and their associated microorganisms will respond to environmental changes. Microbial communities exposed to water stress can produce solutes and extracellular polysaccharides [12, 13] that increase their resistance to water limitations. Since different groups of microbes have different degrees of stress tolerance, long-term exposure to water stress may shift soil microbial profiles towards taxa capable of resisting stress [14–16]. In a laboratory-based experiment, we recently demonstrated that wheat plants had higher root biomass when grown in soil with a history of water stress despite being exposed to the same contemporary water availability as the control group [17]. These results suggest that soil microbes previously subjected to water stress could help plants to cope with subsequent water stresses. However, under lab conditions, wheat plants could not produce sufficient seeds to evaluate the impact of water stress on the seed-associated microbial communities.

Further complexifying the response of plant-associated microorganisms to water stress, plant genotypes with contrasting sensitivity to stress may influence, via rhizodeposition, the recruitment of certain bacteria and fungi in the root environment [18, 19]. Since microbes consume root exudates as carbon sources, plants may change microbial community composition towards more drought-tolerant species [20], which are in turn able to enhance plant responses to drought [21]. Plant-associated microbes also change through the plant developmental stages [22, 23]. For example, the bacterial communities in the rhizosphere of *Arabidopsis thaliana* at the seedling stage differ from the ones at the vegetative, bolting, and flowering stages [23]. The genes related to specific functions (e.g., involved in streptomycin production) were also significantly more expressed at the bolting and flowering stages. However, it is still unclear how microbial stress history and plant genotypes interactively shape the assembly of microbial communities and how this varies across plant compartments and life stages when facing contemporary water stress.

In the present study, we hypothesized that because of soil legacy effects on microbial communities, wheat plants growing in fields with contrasting soil water stress history will be colonized by different microbial communities throughout their compartments, which will be differently affected by contemporary water stress, plant genotype, and growth stages. To test this hypothesis, we grew four wheat genotypes in two agricultural fields (with and without 37 years of water stress history) located side by side, which were then exposed to contemporary water stress (irrigated and non-irrigated) during the whole growing season. We measured the changes in the microbial communities of soil, rhizosphere, roots, and leaves at two distinct stages of plant development, and in and on the seeds at harvest.

## Material and methods

### Field experiment

We made use of two experimental agricultural fields located at Agriculture and Agri-food Canada in Swift Current, SK that are normally used to test the water stress resistance of new wheat varieties. Detailed information about the two agricultural fields is available in Azarbad et al. [17] and Azarbad et al. [24]. The two fields are directly adjacent to one another, located side by side, and have been managed under a continuous wheat-fallow two-year rotation. In semi-arid regions in Canada, such as Saskatchewan, precipitation can be highly variable and limited. Therefore, to maintain soil moisture levels and enhance wheat yields, fallow-wheat or fallow-wheat-wheat has been considered and applied in the farming system [25, 26]. These experimental fields received contrasting irrigation regimes since 1981. They were managed in such a way that one field was irrigated during the growing season of the wheat phase of the rotation (field without water stress history: without WSH) and the other field was not (field with water stress history: with WSH). Another important difference in terms of managing these fields was the differences in fertilization rates. Based on fall soil tests, the fertilizer rates differ each year. For instance, in 2018 when the current experiment was performed, the field without WSH was fertilized with 239 lbs/ac (N:34–P:17), while the field with a WSH received 34 lbs/ac (N:34–P:17). The reason behind the differences in fertilization rate is that the field without WSH had better N utilization (likely due to higher productivity because of the higher soil moisture) and therefore require more fertilizer than the field with WSH. Therefore, every year that wheat was grown in the two fields (every second year based on the rotation), different levels of N and P were used, aiming to bring their concentrations, to some extent, to the same level. The results of soil physicochemical analyses before the start of the experiment (see below), releveled no significant differences in nitrogen levels (NO3-N). However, soil from a field Without WSH contained a higher level of phosphorus concentration than from a field With WSH. A comprehensive analysis of soil chemical and physical characteristics are reported in Supplementary Table S1.

Our experiment started in May 2018. For that, a total of 32 plots (1 m × 3 m) were set up in each of the two fields (with WSH and without WSH). Half of the plots were irrigated in each field, and the other half were non-irrigated. On May 14, 2018, seeds of the four wheat genotypes (two with recognized drought resistance and two without, detailed below) were randomly planted in the experimental plots within each irrigation section, which were arranged in four blocks (blocks were nested within each irrigation treatment). Therefore, this experiment includes 16 treatments: 2 fields × 2 irrigation treatments × 4 wheat genotypes, replicated in 4 blocks (16 × 4 blocks = 64 plots). To characterize soil properties within each field, we used “pre-seeding” soil samples. For that we pooled the samples from plots in each block corresponding to each irrigation treatment, resulting in 16 samples (2 fields × 2 irrigation treatments × 4 blocks). Samples were sent to the A & L Canada Laboratories for soil analyses.

AC Barrie (*Triticum aestivum*) and Strongfield (*Triticum turgidum subsp. durum*) were developed for the semi-arid climatic zone of Canada and known to have drought resistance, whereas AC Nass (*T. aestivum*) and AC Walton (*T. aestivum*) are known to be more productive under well-watered conditions and sensitive to drought. Seeds were purchased from SeCan (Canada’s Seed Partner), one of Canada’s largest suppliers of certified seeds to farmers. Seeds were not subjected to surface sterilization or any chemical or biological products before the start of the experiment, and untreated seeds were used in this study.

The irrigation treatment (25.4 mm at each time point) started on June 19 and continued during the remainder of the growing season on the following dates: June (21 and 28), July (6, 10, 13, 16, 19, 23, 25, 27, and 31) and August (2, 7, and 9). Soil water content was measured before the start of irrigation treatment (May 23, 2018) and 6 weeks after the irrigation treatment started (August 1, 2018). To assess the water status in plants, we measured leaf relative water content (LRWC) at two dates: 2 days after the start of the irrigation treatment (Jun 21, 2018) following 6 weeks after the start of irrigation treatment (August 1, 2018).

Four soil cores from the upper 10 cm were randomly sampled between the crop rows (where the soil was not in direct contact with plants) and categorized as the bulk soil. Soil samples were taken at three dates based on the plant developmental stages as follow: (1) post emergence (May 23, 2018), (2) early stem elongation (June 21, 2018), and (3) early dough development stage (August 1, 2018), for a total of 192 samples (64 plots × 3 developmental stages = 192 samples). Soil cores from the same plot were pooled, homogenized, sieved through a 2.0 mm mesh size sieve, and immediately stored at −20 °C prior to DNA extraction. In addition, we sampled the rhizosphere (soil very firmly attached to the roots), plant leaf, and roots of four random sub-samples per plot at early stem elongation and early dough stages (64 plots × 3 plant compartments × 2 developmental stages = 384 samples). At each sampling point, a group of four wheat plants was collected within each plot manually by digging around the selected plants (up to 50 cm from the soil surface) in order to obtain the entire root system in an intact shape. Rhizosphere sub-samples, soil very firmly attached to the roots, were taken using sterile brushes after the removal of excess soil by shaking the root system of four collected plants. Rhizosphere samples from each plot were mixed, sieved, and stored at −20 °C. In parallel, leaf samples were collected with sterile forceps and stored at −20 °C for downstream analysis. The entire roots were collected and washed on site with sterile water to remove any remaining soil particles and then kept at −20 °C for further procedures. We did our best to sample plants that were to some large extent at the same development stage as detailed above. However, it is important to highlight that the plants from fields with different long-term stress histories were not all at the same physiological stage, likely due to the stress level. For instance, seeds in the field without WSH were harvested on September 11, 2018 and seeds from those plots located in the field with a WSH harvested on August 25, 2018. This took place based on when crops were ready to be harvested. Therefore, 2.5 weeks’ delay in harvest makes sense since plants grown in the field with a WSH were more stressed and matured faster than in a field without WSH. Seed harvest was performed in a careful manner to avoid cross-contamination between irrigated and non-irrigated plots within each field.

Yield (total grain weight for each plot) together with the seed Kernel weight (based on 1000 kernel weight) and protein contents were determined for all the experimental plots. Protein content was measured using a near-infrared analyzer (InfraLUM FT-12 WholeGrain, Mission, Canada). Seeds were kept in plastic bags at room temperature for each plot until DNA extraction. Four non-irrigated plots in the field without WSH had excessive soil moisture content due to slightly lower elevation. Therefore, the entire data set on soil parameters, seed properties, and plant microbiome from these plots were excluded from all analyses.

### DNA extraction, amplicon library preparation, and sequencing

Details on the extraction of the seed endophytes and epiphytes, DNA extraction, amplicon library preparation, sequencing, and bioinformatic analyses are presented in the Supplementary material and methods. Eleven samples of seed endophytes did not amplify successfully for the 16S rRNA gene and were excluded from the sequencing. For the 16S rRNA gene data, out of the 53 seed endophytes samples submitted for sequencing, 32 samples were excluded due to low sequencing depth. Therefore, data on the seed bacterial endophytes were not included in the majority of the statistical models.

### Statistical analyses

All statistical analyses were performed in R (v 4.0.3, The R Foundation for Statistical Computing). We analyzed the data based on linear mixed-effects models (LMM). The lme4 package was used to fit LMMs [27]. LMMs were performed for each field stress history separately. Independent variables (or predictors) in these models were the fixed effects of Irrigation × Genotype × Development stage and the random effect of Block. Block was nested in irrigation. The exception was seeds data where the “Development stage” was not included in the model. Normality and homogeneity of data were assessed based on the visual exploration of plots of residuals versus fitted values. We assessed the statistical significance of fixed predictors using Type II ANOVA including Kenward-Roger methods for denominator degrees of freedom, and of random effects using likelihood ratio tests using the package lmerTest [28]. To investigate how bacterial and fungal α-diversity associated with bulk soil, rhizosphere, root, leaf, seed epiphytes, and seed endophytes were affected by the direct and interactive effect of experimental factors, Shannon’s diversity index was calculated with the otuSummary package [29] and subjected to LMMs. The effect of experimental factors on the bacterial and fungal community structure was visualized using principal coordinate analyses (PCoA) based on Bray–Curtis dissimilarities which was calculated using the vegan package [30]. To test the impact of the experimental parameters and their interactions on bacterial and fungal community structure, PCoA axes 1 and 2 were used in LMMs, similar as in Wagner et al. [31]. In addition, LMMs were performed to determine the impact of experimental factors and their possible interactions on the relative abundance of dominant bacterial and fungal groups at the phyla level within each plant compartment. Following LMMs results, Paired Student’s *t*-test was further used for pairwise comparisons of important bacterial and fungal taxa with respect to the treatments. Finally, ANOVAs, using the aov() function, was used to evaluate the effect of field stress history on soil characteristics.

## Results

### Soil and leaf water contents and quantity and quality of seed

**Fig. 1:**
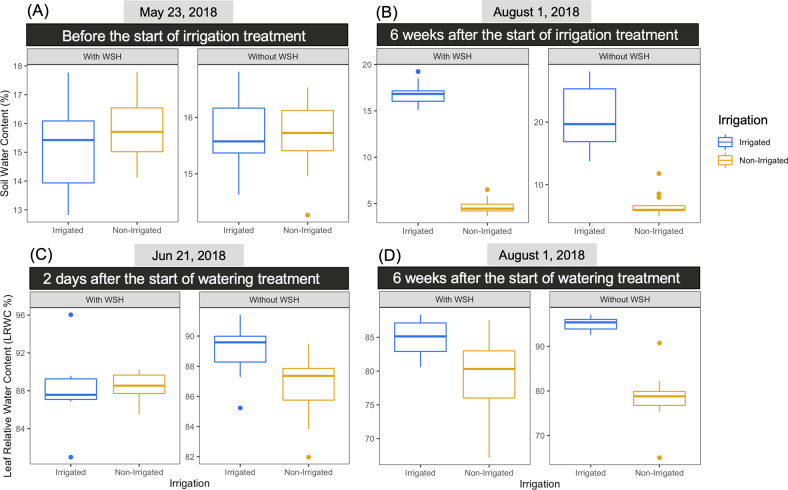
The irrigation treatment significantly changes soil and leaf water contents. Effect of the irrigation treatment (irrigated and non-irrigated) on soil water content (**A** and **B**) and leaf relative water content (**C** and **D**) for four wheat genotypes grown in two agricultural fields with contrasting soil history: without water stress history (without WSH) and with water stress history (with WSH). ANOVA tests are presented in Table S2.

We examined the soil water content (SWC) for all the experimental plots before (May 23, 2018) and 6 weeks after the start of the irrigation treatment (August 1, 2018). As expected, no significant irrigation treatment effects were detected on May 23 (Fig. 1A; Table S2). However, we found that the irrigation treatment had a significant effect on SWC in both fields on August 1, indicating that the irrigation treatments worked successfully (Fig. 1B; Table S2). Six weeks after the start of the irrigation treatment, large increases in LRWC were observed for plants grown in the irrigated plots of the field without WSH (Fig. 1D), but not so much for plants growing in the field with WSH (Fig. 1C). In general, the yields were higher in the field without WSH than in the field with WSH (Fig. S1A). The yields decreased significantly under non-irrigated treatment; however, the extent of the decrease was greatly dependent on field history as wheat grown in the field without WSH exhibited sharper decreases as compared to the wheat grown in the field with a WSH (Fig. S1A; Table 1). In addition, the yields were significantly affected by wheat genotypes and, in general, drought-sensitive genotypes (AC Nass and AC Walton) grown in both fields had higher yields than drought-tolerant genotypes (AC Barrie and Strongfield; Fig. S1A; Table 1). Kernel weight and seed protein content appeared to be primarily impacted by genotype (Figs. S1B and C; Table 1). Independent of field history and irrigation treatment, Strongfield had the highest Kernel weight compared to the other genotypes (Fig. S1B). As for the other genotypes, in both fields, seeds harvested from irrigated plots had in general higher Kernel weight than those harvested from non-irrigated plots. The seed protein content of Strongfield was substantially higher than other genotypes, in particular for seeds harvested from non-irrigated plots. In contrast, AC Nass seeds had the lowest levels of protein in the irrigated plots of the WSH field (Fig. S1C).

**Table 1:** ANOVA tests in LMMs for the effects of irrigation (*I*), genotype (*G*) and their interactions on yield, Kernel weight and seed protein contents for seeds harvested from two agricultural fields with contrasting soil history: without water stress history (without WSH) and with water stress history (with WSH).

	Yield		Kernel weight		Seed protein content	
	With WSH	Without WSH	With WSH	Without WSH	With WSH	Without WSH
*R*^2^	0.86	0.91	0.96	0.93	0.91	0.95
*I*^a^	*F*_1,6_ = 12.3	*F*_1,5_ = 103	*F*_1,6_ = 8.52	F_1,5_ = 36.7	*F*_1,6_ = 3.86	*F*_1,5_ = 25.1
	***P*** = **0.012**	***P*** = **0.000**	***P*** = **0.026**	***P*** = **0.001**	*P* = 096	***P*** = **0.004**
*G*^b^	*F*_3,18_ = 9.50	*F*_3,15_ = 6.319	*F*_3,18_ = 262.4	*F*_3,15_ = 101.9	*F*_3,18_ = 71.1	*F*_3,15_ = 86.5
	***P*** = **0.000**	***P*** = **0.005**	***P*** = **4.86** ^**e-15**^	*P* = **3.34** ^**e-10**^	***P*** = **3.53** ^**e-10**^	***P*** = **1.19** ^**e-09**^
*I* × *G*	*F*_3,18_ = 0.19	*F*_3,15_ = 1.04	*F*_3,18_ = 4.24	*F*_3,15_ = 2.30	*F*_3,18_ = 4.03	*F*_3,15_ = 10.3
	*P* = 0.897	*P* = 0.403	***P*** = **0.019**	*P* = 0.117	***P*** = **0.023**	***P*** = **0.000**
Block	*λ*^2^_1_ = 12.4	*λ*^2^_1_ = 1.686	*λ*^2^_1_ = 2.25	*λ*^2^_1_ = −2.13 ^e-14^	*λ*^2^_1_ = 12.5	*λ*^2^_1_ = 8.08
	***P*** = **0.000**	*P* = 0.194	*P* = 0.133	*P* = 1	***P*** = **0.000**	***P*** = **0.004**

LMMs were performed for each field water stress history separately.

^a^Irrigation treatment refers to irrigation and non-irrigation.

^b^Genotype refers to AC Nass (*Triticum aestivum*), AC Walton (*Triticum aestivum*), AC Barrie (*Triticum aestivum*), Strongfield (*Triticum turgidum* subsp. *durum*).

Bold values indicate statistical significance (*P* < 0.05).

### Bacterial and fungal diversity

Seed-associated epiphytic bacteria from drought-sensitive genotypes (AC Nass and AC Walton) contained higher diversity in comparison to those from drought-tolerant genotypes (AC Barrie and Strongfield) which was only evident in the field with WSH (Table S3; Fig. S2E), indicating an effect of field history. In contrast to bacteria, seeds from drought-tolerant wheat genotypes (AC Barrie and Strongfield) exhibited higher epiphytic fungal diversity than those from drought-sensitive genotypes (AC Nass and AC Walton) (Fig. S4E).

ANOVA tests in LMMs revealed that the development stage significantly impacted bacterial α-diversity associated with the roots, rhizosphere, and leaves (Table S3, Fig. S2). At stem elongation, the rhizosphere of plants grown in the field with WSH had significantly higher bacterial α-diversity as compared to plants at the dough development stage (Fig. S2B). An opposite pattern was observed for plants grown in the field without WSH where at the dough stage the rhizosphere of plants had significantly higher bacterial α-diversity than plants at stem elongation (Fig. S2B). At the dough stage, the roots of plants grown in both fields had significantly higher bacterial diversity as compared with the stem elongation stage (Fig. S2C). In contrast to roots, leaves of plants at stem elongation had in general higher bacterial diversity than at the dough stage, for both fields (Fig. S2D).

The developmental stage also significantly influenced fungal Shannon diversity associated with bulk soil, rhizosphere, roots, and leaves of plants grown in both fields, except for leaves of plants grown in the field with WSH (Table S3; Fig S4). The rhizosphere and roots of plants grown in both fields had significantly higher fungal α-diversity at the dough development stage than at the stem elongation stage which was more evident in the field without WSH (Table S3; Figs. S4B, C). At the stem elongation stage, the leaves of plants grown in the field without WSH contained higher fungal α-diversity than the leaves of plants at the dough developmental stage (Fig. S4D). However, no such pattern was observed for the leaves of plants grown in the field with a WSH, indicating an effect of field water stress history.

### Bacterial and fungal community structures

**Fig. 2:**
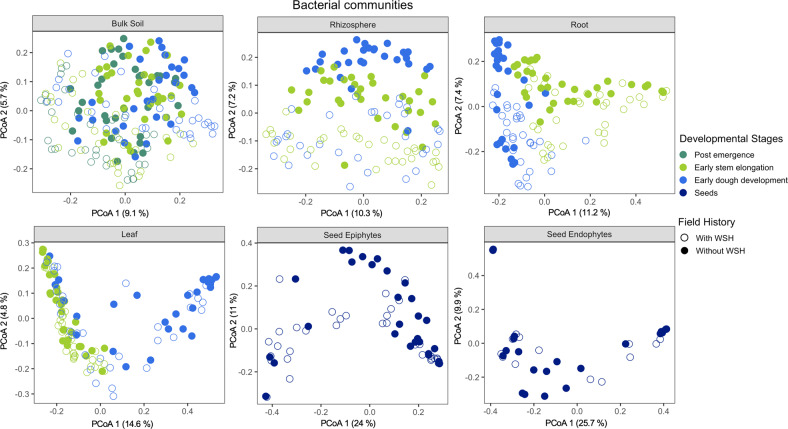
Changes in the bacterial community structure. Principal coordinate analyses (PCoA) of Bray–Curtis dissimilarity to evaluate how dissimilar the bulk soil, rhizosphere, leaf, root, seed epiphytes and endophytes associated bacterial communities were with respect to the experimental factors including irrigation, field history, developmental stages, genotype and their interactions.

**Fig. 3:**
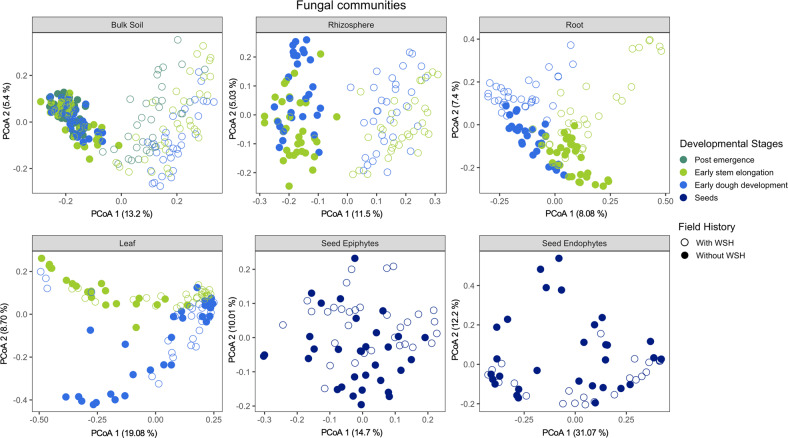
Changes in fungal community structure. Principal coordinate analyses (PCoA) of Bray–Curtis dissimilarity to evaluate how dissimilar the bulk soil, rhizosphere, leaf, root, seed epiphytes and endophytes associated fungal communities were with respect to the experimental factors including irrigation, field history, developmental stages, genotype and their interactions.

We performed principal coordinate analyses (PCoA) to visualize the effects of the experimental factors on the bacterial and fungal community structures for each plant and soil compartments. These results revealed that each plant compartment had distinct bacterial (Fig. S6A) and fungal (Fig. S6B) communities. As shown in the PCoA plots, the seed epi- and endophytic bacterial and fungal communities clustered more closely to the leaves than other plant parts (Fig. S6A, B), indicating similarities between seed and leaves microbial communities. Because of the strong effect of the plant compartment on bacterial and fungal communities, the PCoAs were also performed separately for each compartment (Figs. 2 and 3). Field water stress history and plant developmental stages were the main driving factors shaping bacterial (Fig. 2) and fungal (Fig. 3) communities for most compartments. Linear mixed model analyses of PCoA axes 1 and 2 showed that depending on the field history, the developmental stage had significant direct impacts on one or both PCoA axes for bacterial (Table S4) and fungal (Table S5) profiles for all compartments, except for the seeds.

In the case of the seed bacterial epiphytes, the first PCoA axis separated the communities by field history along with a gradient from irrigated to non-irrigated treatments across the second PCoA axis (Fig. S7). Seed fungal epiphytes from the two different fields were, to a certain extent, separated along the second axis of the PCoA (Fig. 3). The seed epiphytic fungal profile linked with the drought-tolerant genotypes was to some extent separated from the seed of drought-sensitive genotypes along the second axis (Fig. S10) and, in general, seeds harvested from the field without WSH were more profoundly affected by genotype (Table S5).

The rhizosphere of plants grown in both fields was clearly separated along the second (for bacteria; Fig. 2) or first (for fungi; Fig. 3) axis of the PCoA. For the first PCoA of bacterial and the second PCoA of fungal profiles, the direct effects of irrigation were only significant for the rhizosphere of plants grown in the field without WSH (Tables S3 and S4; Figs. S7 and S9). Bacterial and fungal communities associated with the rhizosphere of plants grown in the field with WSH changed less in response to irrigation and the effect of irrigation was only significant for the second PCoA axis of bacterial communities (Tables S4 and S5). In both fields, a more pronounced effect of irrigation was observed for the bacterial profile associated with the leaves at the dough stage (significant interactive effect of Irrigation and Development stage on the first PCoA axis; Table S4). In addition, the direct effect of irrigation was apparent for the first and second PCoA axes of the leaf-associated fungal profiles of plants grown in the field without WSH (Table S5; Fig. S9).

### Bacterial and fungal community composition

**Fig. 4:**
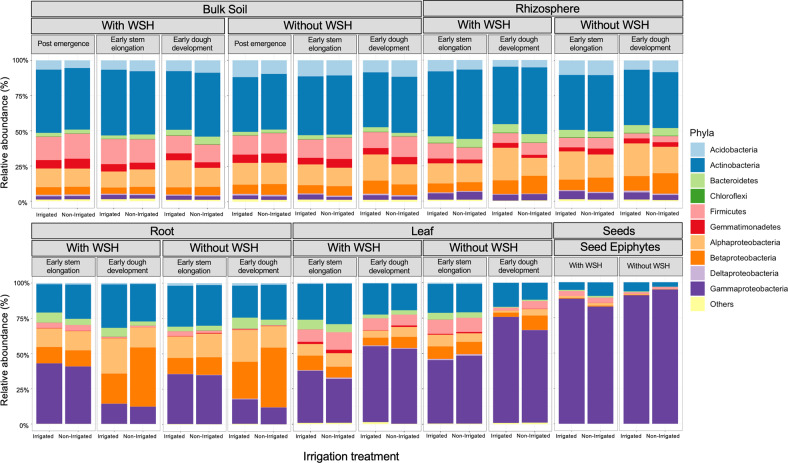
Relative abundance of the most abundant bacterial phyla (at the classes level for *Proteobacteria*) associated with the bulk soil, rhizosphere, leaf, root and seed epiphytes of four wheat genotypes grown in fields without water stress history (without WSH) and with water stress history (with WSH) exposed to irrigation treatment (irrigated and non-irrigated). Developmental stages refer to post emergence, early stem elongation and early dough. Values represent the average of four replicates.

We examined changes in microbial community composition at the phyla/class (bacterial: Fig. 4; fungal: Fig. S12) and orders (bacterial: Fig. S11; fungal: Fig. S13) levels. One striking observation was that throughout all development stages the proportion of *Gammaproteobacteria* was significantly higher in the roots, leaves, and seeds than in the rhizosphere and bulk soils (Fig. 4). The impact of field history was also obvious for many bacterial phyla/classes associated with different compartments (Fig. 4). For example, the relative abundance of *Actinobacteria* in bulk soil, rhizosphere, and leaves from the field with WSH was significantly higher (p < 0.001, *t*-test) than for the field without WSH (Fig. 4). In the case of seed bacterial epiphytes, the field with WSH had a higher relative abundance of *Firmicutes* (*p* = 0.007, *t*-test), linked mostly to the order *Bacillales* (Fig. S11), as compared to the field without WSH (Fig. 4). The same pattern was observed for the rhizosphere samples (*p* < 0.001, *t*-test; Fig. 4; Fig. S11). For fungi, seeds harvested from the field without WSH had a higher relative abundance of epiphytic *Ascomycota* than the seeds harvested from the field with WSH (*p* = 0.005, *t*-test; Fig. S12). For instance, at the order level of this phyla, the relative abundance of *Pleosporales* was significantly higher in the field without WSH than with WSH (*p* < 0.001, *t*-test; Fig. S13). The opposite pattern was observed for epiphytic *Basidiomycota* (*p* = 0.005, *t*-test; Fig. S12).

LMMs identified a significant impact of the developmental stage on the relative abundance of *Firmicutes* associated with the rhizosphere, roots (*p* < 0.001, *t*-test), and leaves (*p* = 0.002, *t*-test) (Fig. 4). In addition, the relative abundance of *Actinobacteria* in the rhizosphere of plants grown in the field with WSH was significantly higher under non-irrigated than irrigated treatment and this pattern was more pronounced at the dough stage (significant interactive effect of Irrigation and Development stage in Table S6; Fig. 4). However, a such pattern was not observed in the rhizosphere of plants grown in the field without WSH (Fig. 4).

In the rhizosphere and roots at stem elongation, the relative abundance of *Zygomycota* was significantly higher than at the dough stage (*p* < 0.001, *t*-test; Fig. S12). The relative abundance of *Ascomycota* in the rhizosphere of plants grown in the field without WSH was significantly higher under non-irrigated than irrigated treatment but only at the dough stage (significant interactive effect of Irrigation and Development stage in Table S7; Fig. S12). For example, at the order level of this phyla, the relative abundance of *Dothideomycetes, Coniochaetales* and *Sordariales* were significantly higher in the field without WSH under non-irrigated than irrigated treatment at the dough stage (Fig. S13). The leaves of plants grown in the field without WSH had a significantly higher relative abundance of *Ascomycota* under the non-irrigated treatment as compared to the irrigated treatment at both developmental stages (Table S7; Fig. S12).

## Discussion

Previous work from our team had used the soil from the current experiment but in a growth room pot study. This had provided us with many insights such as that 37 years of water stress history of the soil microbiome had a constraining effect on bacterial community composition associated with the rhizosphere, root, and leaf when wheat plants were exposed to short-term water stress [24]. However, under these conditions, wheat plants could not produce sufficient seeds to evaluate their microbiota. We replicated our experimental design directly in the field, where we could produce seeds and evaluate if the conclusion from the growth room experiment could be transposed in the field, where the plants were subjected to wider variations in environmental and microbiological conditions. We showed that the plant development stage and field water stress history were the main factors shaping the microbiome associated with all plant compartments. To the best of our knowledge, our results showed for the first time that soil water stress history also impacts bacterial and fungal seed epiphytic communities.

Our results revealed that, within a single generation, seeds can become colonized by divergent microbial communities based on the initial pool of microbes as determined by historical contingencies, but that this is also modulated by the contemporary environmental conditions and the plant genotype. The observed differences between the seed and plant microbial communities from the field with different water stress histories are in line with the previous observations showing that soil microorganisms play important roles in shaping and colonizing the plant microbiota [32–34]. For instance, by inoculating seeds of a single wheat cultivar with soil microbes extracted from 219 soil samples (collected from various soil types across the United States), Walsh et al. showed that soil bacterial communities played critical roles in shaping the bacterial composition of the wheat in the seedling stage [34]. In addition, the interactive effect of irrigation x WSH was observed in many of the seed microbial parameters measured, such as the relative abundance of two key microbial taxa associated with the seed epiphytes, the *Actinobacteria*, and the *Gammaproteobacteria*. These findings are consistent with a recent report by Morales et al. [35] that showed that environmental conditions such as geographical locations and fluctuations in precipitation were the main factors impacting epiphytic bacterial and fungal communities associated with the seeds of eight spring *Brassica napus* when seeds were harvested from different agricultural sites in Saskatchewan, Canada in 2016 and 2017. Seed-associated microorganisms are thought to be the preferred source of microorganisms for developing plants [10] and that plant-associated microbes can impact the host phenotype [7]. Therefore, the critical next step would be to test if the observed water stress-mediated changes in the seed epiphytic and endophytic microbiomes would be beneficial to the next generation of host plants under stress conditions. In addition, we were able to demonstrate that seed endophytic bacteria from drought-sensitive genotypes were more diversified than the ones on drought-tolerant genotypes. In contrast, seeds from drought-tolerant genotypes exhibited higher epiphytic fungal diversity, suggesting contrasting effects of wheat breeding on fungi and bacteria, which is in agreement with previous observations [36]. We need to highlight that our conclusion on the seed microbiome is mainly based on the seed epiphytic bacterial and fungal communities and the hypothesis that contemporary and historical soil water stresses would influence the seed endophytes could not be confirmed due to a lack of sequencing data. While we acknowledge that our data on the seed endophytes is limited, seeds microbiome results bring some first empirical evidence on the effects of adverse environmental conditions on the seed microbiome.

Under well-watered conditions, plants growing in the field without a water stress history had a higher leaf relative water content and higher yields than the ones grown in the field with a water stress history. At the same time, samples from the field without WSH tended to have higher fungal α-diversity as compared to the field with WSH. It has been shown that certain groups of fungi have the capability to enhance plant physiological parameters such as leaf water content [37, 38] and ultimately plant growth and yield [39] under adverse environmental conditions such as drought. Several important caveats need to be kept in mind when interpreting the results of our study. First, as highlighted in the material and methods, the field stress history treatment is based on soil from two fields, a unique setup that is not easily replicable. Thus, it is unfortunate that the effect of soil history could not be directly tested, but to perform this experiment, there were no other ways around this. Therefore, we highly encourage further studies to investigate more soils with different stress histories, which would help to confirm the results of the present study. Second, the experimental fields were fertilized at two different rates (a higher rate of fertilization was applied in the field without WSH). Therefore, different rates of fertilization together with soil abiotic factors which changed over the course of 37 years of contrasting irrigation may explain some of the observed patterns in microbial and plant data. For example, the field without WSH appeared to have a higher level of organic matter, phosphorus, silt, and clay contents than the field with WSH. Most importantly, however, values of soil pH and cation exchange capacity (CEC) which are critical for microbes and plant nutrient availability and uptake were at the same level in both fields. On top of that, this differential fertilization did not appear to clearly influence the grain protein content, and, in fact, the protein content was higher in seeds harvested from the field with WSH (lower N fertilization rate), suggesting that N was not limiting even if applied at a lower rate. Indeed, soil nitrogen level was statistically identical for both fields.

We know from previous studies that different groups of bacterial and fungal taxa may show different response patterns to the water limitation [40, 41], which can eventually shape community structure. In addition, depending on plant genotype and growth stage of the plant, drought can also influence plant biomass which can, in turn, alter belowground C input and eventually the plant microbiome. Our study advances the current understanding in the stress ecology of microbiomes by demonstrating that the impact of contemporary water stress on plants and their microbiome under field conditions are largely dependent on the previous history of soil water stress and plant developmental stages. In particular, our findings indicate that historical water stress conditions influenced the abundance of specific microbial species. For example, the abundance of *Actinobacteria* was higher in the field with WSH than in the field without WSH. These findings agree with previous observations of the impact of drought on the root-associated microbial communities of four rice varieties [42] and could be linked to the capability of *Actinobacteria* to produce stronger cell walls [12] and spores [43]. *Bacillales* (*Firmicutes*), many of which are known plant growth-promoting bacteria [44, 45], were also more abundant on seeds from the field with WSH as compared to the field without WSH and were previously reported to be enriched under dry conditions [42, 46].

The developmental stage significantly shaped microbes associated with the plant tissues, as previously reported for broomcorn millet (*Panicum miliaceum* L.) grown under water stress [47]. It has been shown that these successional patterns are due to changes in root exudation as the plant ages [23]. Similar mechanisms could be at play to explain the observed effects of genotype, especially in leaves, with our recent study that showed the importance of fungal recruitment by the host plants (in particular in the leaves) under water stress [24]. In addition, when discussing how microbiomes associated with different wheat genotypes respond to water stress, it is important to keep in mind that drought resilience is only one trait and other plant characteristics need to be taken into account. For instance, Strongfield is a durum wheat which is susceptible to smut. On the other hand, AC Barrie is a spring wheat known for its resistance to smut. Therefore, the species variation in genotypes used in this study and other traits rather than drought tolerance may, to some certain extent, explain the observed significant interaction effects between genotype and other experimental variables on the measured plant and microbiome parameters.

In conclusion, our results demonstrate that historical and contemporary soil water stress, genotype, and developmental stage of plants can have interactive effects on bacterial and fungal communities from all major plant compartments. Further, we showed that historical environmental conditions can alter the seed microbiome. The next step would be to test if these effects are transferred through the seeds to influence the growth of the next generation of plants under water stress conditions.

## Supplementary information

Supplementary material and methods

Supplementary Tables

## Data Availability

The data that supports the findings of this study are available in the supplementary material of this article. Raw data sets are available in the NCBI Sequence Read Archive (SRA) under the BioProject accession PRJNA736197.
