# Supplementary material for: The response of wheat and its microbiome to contemporary and historical water stress in a field experiment
Source: ISME Commun. 2022 Jul 27;2:62. doi: 10.1038/s43705-022-00151-2 (PMC9723694; doi:10.1038/s43705-022-00151-2)
Supplement: Supplementary file 1 — Supplementary material and methods [file 43705_2022_151_MOESM1_ESM.docx]

**The response of wheat and its microbiome to contemporary and historical water stress** **in a field experiment**

Hamed Azarbad^1*^, Luke D. Bainard^2^, Asmaâ Agoussar^3^, Julien Tremblay^4^, and Etienne Yergeau^3^

**Supplementary material and methods**

*Extracting the seed endophytes and epiphytes microbes*

For the seed endophytes extraction (total of 64 samples), peptone buffer solution (10 g peptone, 5 g sodium chloride (NaCl), 3.5 g di-sodium hydrogen phosphate (Na_2_HPO_4_), 1.5 g of potassium di-hydrogen phosphate (KH_2_PO_4_) per liter) was prepared as described previously by Kim et al. (2006) with some modifications. Then, 10 g of seed corresponding to each of 64 plots were added into a 250 ml sterile Erlenmeyer flask containing 45 ml of autoclaved peptone buffer (freshly made). The Erlenmeyer flasks were carefully placed on a shaker for 1 h at 150 rpm. The liquid fractions were then centrifuged at 4000 g for 15 min and the pellets were resuspended in 1000 μl of TEP buffer (10 mM Tris-HCl (pH 8.0) and 1.0 mM EDTA (pH 8.0)) and stored at −20°C in the laboratory prior to DNA extraction. For endophytes extraction, the seed samples from the previous part were subjected to surface sterilization after extraction of epiphytic microbe following the protocol described by Sun et al. (2008), with some modifications. Briefly, wheat seeds were washed with sterile water and immersed in 95 % ethanol for 5 min. They were then washed with sodium hypochlorite solution (2.5 % available Cl^−^) for 5 min, rinsed with 95 % alcohol for 3 min, and finally washed 3 more times with sterile water. To confirm if the sterilization of the surface of seeds was successful and that no seed surface bacteria and fungi remained in the solution, 100 μl of the final rinsing water were spread on Tryptic Soybean Broth (TSB) solid medium plates and cultured for 3 days at 28 °C. No microbial growth was noted. Then the liquid nitrogen with mortar and pestle was used to immediately crush the seed sample. In the final stage, 0.5 g of each sample was kept at −20 °C for DNA extraction of endophytic microbes.

*DNA extraction, amplicon library preparation and sequencing*

For DNA extraction, leaf and root samples were ground in powder using liquid nitrogen with a mortar and pestle. DNA was extracted from 0.5 g of bulk soil, rhizosphere, roots, leaves and the seed (endophyte) samples using a phenol-chloroform extraction method (Dellaporta *et al*., 1983). For seed epiphyte extraction, pellets which were resuspended in 1000 μl of TEP buffer were used directly for phenol-chloroform DNA extraction. A total of 704 samples were extracted. Detailed information on DNA extraction is presented in Azarbad *et al*. (2018). Sequencing libraries were prepared using two steps approach as described previously (Yergeau *et al*., 2015). Amplicon libraries were prepared for the bacterial 16S rRNA gene using the universal primers 520F and 799R targeting the V4 region (Edwards *et al*., 2007) and for the fungal ITS1 region using ITS1F and 58A2R primers (Martin and Rygiewicz, 2005). Samples correspond to the bacteria and fungi were pooled separately and sequenced on an Illumina MiSeq sequencer (250PE) at the Centre d’expertise et de séquençage Genome Québec (Montréal, Canada).

*Analysis of sequencing data*

Sequences were analyzed using AmpliconTagger (Tremblay and Yergeau, 2019). Briefly, raw reads were scanned for sequencing adapters and PhiX spike-in sequences. The remaining reads were filtered based on quality (Phred) score and remaining sequences were dereplicated/clustered at 100% identity and then processed for generating Amplicon Sequence Variants (ASVs) (DADA2 v1.12.1) (PMID:27214047). Chimeras were removed with DADA2’s internal removeBimeraDeNovo (method= “consensus”) method followed by UCHIME reference (Edgar et al., 2011). ASVs for which abundance across all samples were lower than 3 were discarded. ASVs were assigned a taxonomic lineage with the RDP classifier (PMID: 17586664) using an in-house training set containing the complete Silva release 128 database (PMID:23193283) supplemented with eukaryotic sequences from the Silva database and a customized set of mitochondria, plasmid and bacterial 16S sequences. For ITS ASVs, a training set containing the Unite DB was used (sh_general_release_s_04.02.2020 version). The RDP classifier assigns a score (0 to 1) to each taxonomic depth of each ASV. Each taxonomic depth having a score ≥ 0.5 were kept to reconstruct the final lineage. Taxonomic lineages were combined with the cluster abundance matrix obtained above to generate a raw ASV table, from which a bacterial organisms ASV table was generated. Taxonomic summaries were computed using the QIIME v1.9.1 software suite (PMID: 20383131, 22161565) using the ASV table of each data type.

**References**

**Azarbad H, Constant P, Giard-Laliberté C, Bainard LD, Yergeau E**. **2018**. Water stress history and wheat genotype modulate rhizosphere microbial response to drought. *Soil Biology and Biochemistry* **126**: 228–236.

**Dellaporta SL, Wood J, Hicks JB**. **1983**. A plant DNA minipreparation: Version II. *Plant Molecular Biology Reporter* **1**: 19–21.

**Edwards JE, Huws SA, Kim EJ, Kingston-Smith AH**. **2007**. Characterization of the dynamics of initial bacterial colonization of nonconserved forage in the bovine rumen. *FEMS microbiology ecology* **62**: 323–335.

**Kim J, Demeke T, Clear RM, Patrick SK**. **2006**. Simultaneous detection by PCR of Escherichia coli, Listeria monocytogenes and Salmonella typhimurium in artificially inoculated wheat grain. *International Journal of Food Microbiology* **111**: 21–25.

**Kõljalg U, Nilsson RH, Abarenkov K, Tedersoo L, Taylor AFS, Bahram M, Bates ST, Bruns TD, Bengtsson-Palme J, Callaghan TM, *et al.*** **2013**. Towards a unified paradigm for sequence-based identification of fungi. *Molecular Ecology* **22**: 5271–5277.

**Martin KJ, Rygiewicz PT**. **2005**. Fungal-specific PCR primers developed for analysis of the ITS region of environmental DNA extracts. *BMC Microbiology* **5**: 28.

**Sun L, Qiu F, Zhang X, Dai X, Dong X, Song W**. **2008**. Endophytic bacterial diversity in rice (Oryza sativa L.) roots estimated by 16S rDNA sequence analysis. *Microbial Ecology* **55**: 415–424.

**Tremblay J, Yergeau E.** 2019. Systematic Processing of Ribosomal RNA Gene Amplicon Sequencing Data. *GigaScience* 8 (12).

**Yergeau E, Bell TH, Champagne J, Maynard C, Tardif S, Tremblay J, Greer CW**. **2015**. Transplanting Soil Microbiomes Leads to Lasting Effects on Willow Growth, but not on the Rhizosphere Microbiome. *Frontiers in Microbiology* **6**: 1436.
