# Supplementary material for: The response of wheat and its microbiome to contemporary and historical water stress in a field experiment
Source: ISME Commun. 2022 Jul 27;2:62. doi: 10.1038/s43705-022-00151-2 (PMC9723694; doi:10.1038/s43705-022-00151-2)
Supplement: Supplementary file 2 — Supplementary Tables [file 43705_2022_151_MOESM2_ESM.docx]

**Table S1.** Soil physicochemical analyses (means and standard deviations (STD), n = 8) of two agricultural fields with contrasting soil history: without water stress history (without WSH) and with water stress history (with WSH). ANOVA was used for the comparison of soil parameters between two fields, taking into account the block effect.

|  | **Without WSH** | | **With WSH** | | Field effect | Block effect |
| --- | --- | --- | --- | --- | --- | --- |
| Soil characteristics | Means | STD | Means | STD | P value | P value |
| Organic Matter | 2.86 | 0.11 | 2.61 | 0.06 | **<0.001** | 0.325 |
| pH (water) | 5.25 | 0.16 | 5.28 | 0.19 | 0.726 | **0.009** |
| pH (CaCl_2_) | 6.33 | 0.17 | 6.40 | 0.16 | 0.334 | 0.073 |
| P (ppm)_bicarbonate method | 32.1 | 4.58 | 25.6 | 4.75 | **0.017** | 0.522 |
| P (ppm)_Bray-1 method | 62.1 | 6.51 | 48.4 | 6.84 | **0.001** | 0.502 |
| NO3-N (ppm) | 28.0 | 5.78 | 23.1 | 5.41 | 0.117 | 0.925 |
| Na (ppm) | 33.13 | 9.54 | 18.6 | 1.06 | **<0.001** | 0.222 |
| K (ppm) | 370.7 | 48.4 | 318 | 46.9 | **0.046** | 0.358 |
| Mg (ppm) | 314.3 | 25.0 | 308.7 | 33.1 | 0.530 | **<0.001** |
| Ca (ppm) | 1196 | 76.2 | 1317 | 97.0 | **0.007** | **0.038** |
| Al (ppm) | 865.3 | 50.3 | 778.1 | 53.2 | **0.005** | 0.437 |
| PBS_P (%) ^a^ | 9.25 | 0.46 | 8.00 | 0.76 | **0.001** | 0.160 |
| PBS_Na (%) | 0.80 | 0.18 | 0.46 | 0.05 | **<0.001** | 0.069 |
| PBS_K (%) | 5.38 | 0.76 | 4.71 | 0.47 | 0.064 | 0.801 |
| PBS_Mg (%) | 14.9 | 2.39 | 15.1 | 2.72 | 0.877 | **0.012** |
| PBS_Ca (%) | 34.0 | 4.67 | 38.6 | 5.49 | 0.069 | 0.065 |
| PBS_Al (%) | 1.43 | 0.31 | 1.30 | 0.38 | 0.393 | **0.016** |
| PBS_H (%) | 44.8 | 7.24 | 41.0 | 7.99 | 0.280 | **0.037** |
| K/Mg Ratio | 0.36 | 0.06 | 0.32 | 0.08 | 0.189 | **0.023** |
| CEC (meq/100g) ^b^ | 17.4 | 1.83 | 17.2 | 1.45 | 0.858 | 0.327 |
| ENR ^c^ | 40.6 | 1.06 | 38.1 | 0.64 | **<0.001** | 0.325 |
| Sand (%) | 54.7 | 1.58 | 58.7 | 3.41 | **0.012** | 0.750 |
| Silt (%) | 33.4 | 0.92 | 30.6 | 2.39 | **0.011** | 0.638 |
| Clay (%) | 12.0 | 0.76 | 10.7 | 0.89 | **0.010** | 0.435 |

^a^ Percent base saturation (PBS), ^b^ Cation exchange capacity (CEC), ^c^ Estimated Nitrogen Release (ENR) from the soil.

**Table S2.** ANOVA tests in LMMs for the effects of irrigation (I), genotype (G) and their interactions on soil and leaf water contents. LMMs was performed for each field water stress history separately.

| Soil water contents | | | | | |
| --- | --- | --- | --- | --- | --- |
|  | SWC (May 23, 2018) | | | SWC (August 1, 2018) | |
|  | With WSH | Without WSH | With WSH | | Without WSH |
| R^2^ | 0.55 | 0.01 | 0.98 | | 0.95 |
| I ^a^ | F _1,6_ = 0.56  P = 0.480 | F _1,5_ = 0.16  P = 0.703 | F _1,6_ = 727  **P = 1.71 ^e-7^** | | F _1,5_ = 19.0  **P = 0.007** |
| G ^b^ | F _3,18_ = 0.21  P = 0.887 | F _3,15_ = 1.62  P = 0.225 | F _3,18_ = 0.52  P = 0.671 | | F _3,15_ = 0.24  P = 0.864 |
| I × G | F _3,18_ = 0.66  P = 0.584 | F _3,15_ = 1.13  P = 0.367 | F _3,18_ = 0.14  P = 0.931 | | F _3,15_ = 0.93  P = 0.448 |
| Block | λ^2^ _1_ = 6.57  **P = 0.010** | λ^2^ _1_ = -4.9^e-14^  P = 1 | λ^2^ _1_ = 1.03  P = 0.304 | | λ^2^ _1_ = 19.6  P = **9.4 ^e-6^** |
|  | | | | | |
| Leaf water contents | | | | | |
|  | LRWC (Jun 21, 2018) | | | LRWC (August 1, 2018) | |
|  | With WSH | Without WSH | With WSH | | Without WSH |
| R^2^ | 0.23 | 0.55 | 0.47 | | 0.82 |
| I | F _1,6_ = 0.29  P = 0.589 | F _1,5_ = 5.96  P = 0.058 | F _1,6_ = 16.2  **P = 0.006** | | F _1,5_ = 117  **P = 0.000** |
| G | F _3,18_ = 2.05  P = 0.132 | F _3,15_ = 1.05  P = 0.398 | F _3,18_ = 1.74  P = 0.194 | | F _3,15_ = 0.69  P = 0.570 |
| I × G | F _3,18_ = 0.87  P = 0.466 | F _3,15_ = 0.54  P = 0.656 | F _3,18_ = 1.96  P = 0.55 | | F _3,15_ = 0.87  P = 0.473 |
| Block | λ^2^ _1_ = -8.52 ^e-14^  P = 1 | λ^2^ _1_ = 2.35  P = 0.124 | λ^2^ _1_ = 0  P = 1 | | λ^2^ _1_ = 0  P = 1 |

^a^ Irrigation treatment refers to irrigation and non-irrigation.

^b^ Genotype refers to AC Nass (*Triticum aestivum*), AC Walton (*Triticum aestivum*), AC Barrie (*Triticum aestivum*), Strongfield (*Triticum turgidum* subsp. *durum*)

| Bacteria Shannon diversity | | | | | | | | | | | | |
| --- | --- | --- | --- | --- | --- | --- | --- | --- | --- | --- | --- | --- |
|  | Bulk soil | | Rhizosphere | | Root | | Leaf | | Seed Epiphytes | | Seed Endophytes | |
|  | With  WSH | Without  WSH | With  WSH | Without  WSH | With  WSH | Without  WSH | With  WSH | Without  WSH | With  WSH | Without  WSH | With  WSH | Without  WSH |
| R^2^ | 0.18 | 0.17 | 0.29 | 0.38 | 0.40 | 0.32 | 0.42 | 0.56 | 0.35 | 0.29 | - | - |
| I | F _1,6_ = 0.16  P = 0.696 | F _1,5_ = 0.02  P = 0.880 | F _1,5_ = 0.01  P = 0.912 | F _1,5_ = 0.63  P = 0.458 | F _1,6_ = 1.92  P = 0.214 | F _1,5_ = 1.12  P = 0.331 | F _1,5_ = 0.01  P = 0.903 | F _1,5_ = 0.97  P = 0.362 | F _1,5_ = 0.35  P = 0.575 | F _1,5_ = 0.01  P = 0.912 | - | - |
| G | F _3,66_ = 1.03  P = 0.383 | F _3,62_ = 0.21  P = 0.888 | F _3,41_ = 1.1  P = 0.359 | F _3,37_ = 1.05  P = 0.378 | F _3,42_ = 0.99  P = 0.404 | F _3,38_ = 0.89  P = 0.454 | F _3,40_ = 2.7  P = 0.056 | F _3,35_ = 0.94  P = 0.427 | F _3,17_ = 4.01  **P = 0.024** | F _3,17_ = 0.68  P = 0.575 | - | - |
| D | F _2,66_ = 1.98  P = 0.145 | F _2,63_ = 2.48  P = 0.091 | F _1,41_ = 5.9  **P = 0.019** | F _1,38_ = 19.7  **P = 0.000** | F _1,42_ = 17.6  **P = 0.000** | F _1,40_ = 7.56  **P = 0.008** | F _1,40_ = 21.7  **P = 0.000** | F _1,36_ = 42.6  **P = 0.000** | - | - | - | - |
| I × G | F _3,66_ = 0.09  P = 0.961 | F _3,62_ = 0.49  P = 0.687 | F _3,41_ = 0.42  P = 0.735 | F _3,37_ = 1.67  P = 0.189 | F _3,42_ = 1.28  P = 0.293 | F _3,38_ = 2.55  P = 0.069 | F _3,40_ = 1.12  P = 0.348 | F _3,35_ = 0.60  P = 0.613 | F _3,17_ = 1.11  P = 0.370 | F _3,17_ = 1.73  P = 0.19 | - | - |
| I × D | F _2,66_ = 1.89  P = 0.157 | F _2,63_ = 0.87  P = 0.420 | F _1,41_ = 0.04  P = 0.831 | F _1,38_ = 0.00  P = 0.922 | F _1,42_ = 3.76  P = 0.059 | F _1,41_ = 0.69  P = 0.407 | F _1,40_ = 0.28  P = 0.597 | F _1,37_ = 4.60  **P = 0.038** | - | - | - | - |
| G × D | F _6,66_ = 0.72  P = 0.631 | F _6,62_ = 0.40  P = 0.875 | F _3,41_ = 3.40  **P = 0.026** | F _3,37_ = 0.47  P = 0.704 | F _3,42_ = 0.69  P = 0.562 | F _3,38_ = 0.70  P = 0.552 | F _3,40_ = 2.89  **P = 0.046** | F _3,35_ = 1.54  P = 0.219 | - | - | - | - |
| I × G × D | F _6,66_ = 0.80  P = 0.572 | F _6,62_ = 1.22  P = 0.303 | F _3,41_ = 0.41  P = 0.742 | F _3,37_ = 0.18  P = 0.908 | F _3,42_ = 1.02  P = 0.393 | F _3,38_ = 1.41  P = 0.254 | F _3,40_ = 0.08  P = 0.969 | F _3,35_ = 0.71  P = 0.548 | - | - | - | - |
| Block | λ^2^ _1_ = 1.4 ^e-14^  P = 1 | λ^2^ _1_ = 8.2 ^e-14^  P = 1 | λ^2^ _1_ = 0.29  P = 0.584 | λ^2^ _1_ = 0.45  P = 0.501 | λ^2^ _1_ = 0.63  P = 0.423 | λ^2^ _1_ = 5.6 ^e-14^  P = 1 | λ^2^ _1_ = 2.8 ^e-14^  P = 1 | λ^2^ _1_ = 1.16  P = 0.280 | λ^2^ _1_ = 1.4 ^e-14^  P = 1 | λ^2^ _1_ = 0.54  P = 0.461 | - | - |
|  | | | | | | | | | | | | |
| Fungi Shannon diversity | | | | | | | | | | | | |
|  | Bulk soil | | Rhizosphere | | Root | | Leaf | | Seed Epiphytes | | Seed Endophytes | |
|  | With  WSH | Without  WSH | With  WSH | Without  WSH | With  WSH | Without  WSH | With  WSH | Without  WSH | With  WSH | Without  WSH | With  WSH | Without  WSH |
| R^2^ | 0.29 | 0.44 | 0.16 | 0.56 | 0.44 | 0.25 | 0.37 | 0.41 | 0.40 | 0.59 | 0.30 | 0.41 |
| I | F _1,6_ = 5.83  **P = 0.052** | F _1,5_ = 5.37  P = 0.060 | F _1,5_ = 0.58  P = 0.472 | F _1,5_ = 4.67  P = 0.076 | F _1,5_ = 0.20  P = 0.664 | F _1,5_ = 0.76  P = 0.418 | F _1,5_ = 0.59  P = 0.469 | F _1,4_ = 1.17  P = 0.328 | F _1,6_ = 2.22  P = 0.186 | F _1,6_ = 0.23  P = 0.642 | F _1,4_ = 0.31  P = 0.606 | F _1,4_ = 0.13  P = 0.726 |
| G | F _3,66_ = 0.37  P = 0.771 | F _3,61_ = 2.42  P = 0.074 | F _3,41_ = 0.41  P = 0.740 | F _3,37_ = 4.49  **P = 0.008** | F _3,41_ = 1.25  P = 0.301 | F _3,37_ = 0.05  P = 0.983 | F _3,37_ = 2.23  P = 0.100 | F _3,27_ = 0.71  P = 0.552 | F _3,18_ = 5.16  **P = 0.009** | F _3,18_ = 10.9  **P = 0.000** | F _3,11_ = 1.11  P = 0.382 | F _3,11_ = 0.46  P = 0.713 |
| D | F _2,66_ = 3.69  **P = 0.030** | F _2,62_ = 8.17  **P = 0.000** | F _1,41_ = 4.67  **P = 0.036** | F _1,38_ = 34.5  **P = 0.000** | F _1,41_ = 20.5  **P = 0.000** | F _1,39_ = 8.86  **P = 0.004** | F _1,37_ = 0.59  P = 0.445 | F _1,27_ = 19.5  **P = 0.000** | - | - | - | - |
| I × G | F _3,66_ = 3.07  **P = 0.033** | F _3,61_ = 1.21  P = 0.311 | F _3,41_ = 0.24  P = 0.867 | F _3,37_ = 3.14  **P = 0.036** | F _3,41_ = 0.46  P = 0.709 | F _3,37_ = 0.28  P = 0.833 | F _3,37_ = 1.89  P = 0.146 | F _3,28_ = 0.36  P = 0.781 | F _3,18_ = 1.09  P = 0.377 | F _3,18_ = 0.95  P = 0.433 | F _3,11_ = 1.86  P = 0.192 | F _3,11_ = 0.17  P = 0.911 |
| I × D | F _2,66_ = 0.88  P = 0.419 | F _2,62_ = 7.45  **P = 0.001** | F _1,41_ = 0.72  P = 0.399 | F _1,38_ = 2.04  P = 0.160 | F _1,41_ = 10.5  **P = 0.002** | F _1,39_ = 0.23  P = 0.629 | F _1,37_ = 0.59  P = 0.444 | F _1,27_ = 1.26  P = 0.270 | - | - | - | - |
| G × D | F _6,66_ = 1.57  P = 0.167 | F _6,61_ = 0.36  P = 0.901 | F _3,41_ = 0.66  P = 0.580 | F _3,37_ = 1.10  P = 0.358 | F _3,41_ = 1.81  P = 0.158 | F _3,37_ = 1.96  P = 0.135 | F _3,37_ = 1.04  P = 0.382 | F _3,29_ = 1.50  P = 0.234 | - | - | - | - |
| I × G × D | F _6,66_ = 0.58  P = 0.741 | F _6,61_ = 2.77  **P = 0.018** | F _3,41_ = 0.54  P = 0.655 | F _3,37_ = 0.28  P = 0.836 | F _3,41_ = 0.77  P = 0.515 | F _3,37_ = 0.08  P = 0.968 | F _3,37_ = 0.32  P = 0.808 | F _3,29_ = 0.74  P = 0.536 | - | - | - | - |
| Block | λ^2^ _1_ = 1.4 ^e-14^  P = 1 | λ^2^ _1_ = 0.11  P = 0.736 | λ^2^ _1_ = 2.8 ^e-14^  P = 1 | λ^2^ _1_ = 0.34  P = 0.558 | λ^2^ _1_ = 0.46  P = 0.494 | λ^2^ _1_ = 0.13  P = 0.709 | λ^2^ _1_ = 3.06  P = 0.080 | λ^2^ _1_ = 0  P = 1 | λ^2^ _1_ = -9.7 ^e-15^  P = 1 | λ^2^ _1_ = 1.22  P = 0.268 | λ^2^ _1_ = -2.8 ^e-14^  P = 1 | λ^2^ _1_ = 3.64  P = 0.056 |

**Table S3.** ANOVA tests in LMMs for the effects of irrigation (I), genotype (G), developmental stages (D) and their interactions on bacteria and fungi Shannon diversity of bulk soil, rhizosphere, leaf, root, seed epiphytes and endophytes communities.

| Bacterial communities | | | | | | | | | | | | |
| --- | --- | --- | --- | --- | --- | --- | --- | --- | --- | --- | --- | --- |
|  | Bulk soil | | | | Rhizosphere | | | | Root | | | |
|  | With WSH | | Without WSH | | With WSH | | Without WSH | | With WSH | | Without WSH | |
|  | PCo1 | PCo2 | PCo1 | PCo2 | PCo1 | PCo2 | PCo1 | PCo2 | PCo1 | PCo2 | PCo1 | PCo2 |
| R^2^ | 0.86 | 0.81 | 0.78 | 0.78 | 0.94 | 0.62 | 0.84 | 0.58 | 0.540 | 0.86 | - | - |
| I | F _1,6_ = 0.00  P = 0.954 | F _1,6_ = 0.02  P = 0.878 | F _1,5_ = 3.65  P = 0.104 | F _1,5_ = 0.23  P = 0.643 | F _1,6_ = 0.02  P = 0.884 | F _1,5_ = 13.2  **P = 0.010** | F _1,5_ = 6.51  **P = 0.043** | F _1,5_ = 0.09  P = 0.774 | F _1,6_ = 1.97  P = 0.209 | F _1,6_ = 0.51 P = 0.498 | F _1,5_ = 0.31  P = 0.598 | F = 16.5  **P = 0.006** |
| G | F _3,66_ = 0.25  P = 0.858 | F _3,66_ = 1.99  P = 0.123 | F _3,62_ = 0.69  P = 0.557 | F _3,62_ = 0.25 P = 0.854 | F _3,41_ = 0.31  P = 0.811 | F _3,41_ = 1.80  P = 0.161 | F _3,38_ = 1.64  P = 0.195 | F _3,38_ = 0.40  P = 0.753 | F _3,42_ = 1.82  P = 0.158 | F _3,42_ = 1.70 P = 0.181 | F _3,38_ = 0.52  P = 0.668 | F = 0.85  P = 0.474 |
| D | F _2,66_ = 52.7  **P = 0.000** | F _2,66_ = 29.0  **P = 0.000** | F _2,62_ = 12.6  **P = 0.000** | F _2,62_ = 0.95  P = 0.390 | F _1,41_ = 1.21  P = 0.277 | F _1,41_ = 8.59  **P = 0.005** | F _1,38_ = 0.15  P = 0.698 | F _1,39_ = 53.6  **P = 0.000** | F _1,42_ = 59.1  **P = 0.000** | F _3,42_ = 74.1 **P = 0.000** | F _1,38_ = 51.1  **P = 0.000** | F = 5.28  **P = 0.027** |
| I × G | F _3,66_ = 0.67  P = 0.571 | F _3,66_ = 1.75  P = 0.164 | F _3,62_ = 2.51  P = 0.066 | F _3,62_ = 4.22  **P = 0.008** | F _3,41_ = 0.31  P = 0.812 | F _3,41_ = 0.66  P = 0.578 | F _3,38_ = 0.39  P = 0.755 | F _3,38_ = 1.37  P = 0.264 | F _3,42_ = 1.06  P = 0.374 | F _3,42_ = 0.10 P = 0.95 | F _3,38_ = 1.79  P = 0.164 | F = 0.26  P = 0.852 |
| I × D | F _2,66_ = 2.73  P = 0.072 | F _2,66_ = 1.45  P = 0.240 | F _2,62_ = 1.84  P = 0.167 | F _2,62_ = 1.57  P = 0.215 | F _1,41_ = 7.60  **P = 0.008** | F _1,41_ = 16.1  **P = 0.000** | F _1,38_ = 5.59  **P = 0.023** | F _1,39_ = 0.09  P = 0.763 | F _1,42_ = 0.09  P = 0.758 | F _3,42_ = 53.4 **P = 0.000** | F _1,39_ = 0.16 P = 0.683 | F = 65.3  **P = 0.000** |
| G × D | F _6,66_ = 0.36  P = 0.896 | F _6,66_ = 0.63  P = 0.702 | F _6,62_ = 1.11  P = 0.363 | F _6,62_ = 0.66  P = 0.680 | F _3,41_ = 0.37  P = 0.773 | F _3,41_ = 3.14  **P = 0.035** | F _3,38_ = 1.77  P = 0.168 | F _3,38_ = 0.55  P = 0.650 | F _3,42_ = 1.00  P = 0.399 | F _3,42_ = 0.86 P = 0.464 | F _3,38_ = 1.19  P = 0.324 | F = 0.66  P = 0.578 |
| I × G × D | F _6,66_ = 0.21  P = 0.972 | F _6,66_ = 0.49  P = 0.812 | F _6,62_ = 0.481  P = 0.819 | F _6,62_ = 0.24  P = 0.960 | F _3,41_ = 0.17  P = 0.909 | F _3,41_ = 0.42  P = 0.734 | F _3,38_ = 2.33  P = 0.088 | F _3,38_ = 0.12  P = 0.943 | F _3,42_ = 0.01 P = 0.998 | F _3,42_ = 0.15 P = 0.922 | F _3,38_ = 1.56  P = 0.214 | F = 0.12  P = 0.947 |
| Block | λ^2^ _1_ = 105  **P = 2.2 ^e-14^** | λ^2^ _1_ = 83  **P = 2.2 ^e-16^** | λ^2^ _1_ = 58.7  **P = 1.7 ^e-14^** | λ^2^ _1_ = 75.9  **P = 2.2 ^e-16^** | λ^2^ _1_ = 104  **P = 2.2 ^e-16^** | λ^2^ _1_ = 4.58  **P = 0.032** | λ^2^ _1_ = 41  **P = 1.4 ^e-10^** | λ^2^ _1_ = 3.88  **P = 0.048** | λ^2^ _1_ = -**2.1 ^e-14^**  P = 1 | λ^2^ _1_ = 54.2  **P = 1.7 ^e-13^** | λ^2^ _1_ = 0.87  P = 0.35 | λ^2^ _1_ = 18.4  **P = 1.7 ^e-05^** |
|  | | | | | | | | | | | | |
|  | Leaf | | | | Seed Epiphytes | | | | Seed Endophytes | | | |
|  | With WSH | | Without WSH | | With WSH | | Without WSH | | With WSH | | Without WSH | |
|  | PCo1 | PCo2 | PCo1 | PCo2 | PCo1 | PCo2 | PCo1 | PCo2 | PCo1 | PCo2 | PCo1 | PCo2 |
| R^2^ | 0.63 | 0.35 | 0.78 | 0.29 | 0.30 | 0.41 | 0.21 | 0.51 | - | - | - | - |
| I | F _1,5_ = 22.0  **P = 0.003** | F _1,5_ = 0.00  P = 0.959 | F _1,5_ = 29.0  **P = 0.001** | F _1,5_ = 0.00  P = 0.994 | F _1,6_ = 2.17  P = 0.190 | F _1,6_ = 4.67  P = 0.073 | F _1,5_ = 0.02  P = 0.872 | F _1,5_ = 2.27  P = 0.150 | - | - | - | - |
| G | F _3,41_ = 2.28  P = 0.092 | F _3,41_ = 5.66  **P = 0.002** | F _3,36_ = 0.24  P = 0.866 | F _3,36_ = 1.15  P = 0.340 | F _3,18_ = 2.69  P = 0.076 | F _3,18_ = 0.74  P = 0.53 | F _3,17_ = 0.81  P = 0.502 | F _3,17_ = 1.76  P = 0.192 | - | - | - | - |
| D | F _1,41_ = 32.7  **P = 0.000** | F _1,41_ = 2.24  P = 0.142 | F _1,38_ = 124.4  **P = 0.000** | F _1,37_ = 0.31  P = 0.577 | - | - | - | - | - | - | - | - |
| I × G | F _3,41_ = 0.67  P = 0.572 | F _3,41_ = 0.84  P = 0.475 | F _3.36_ = 0.71  P = 0.552 | F _3.36_ = 1.69  P = 0.184 | F _3,18_ = 1.07  P = 0.384 | F _3,18_ = 0.71  P = 0.55 | F _3,17_ = 0.63  P = 0.600 | F _3,17_ = 2.37  P = 0.105 | - | - | - | - |
| I × D | F _1,41_ = 30.3  **P = 0.000** | F _1,41_ = 2.00  P = 0.164 | F _1,38_ = 30.0  **P = 0.000** | F _1,38_ = 0.48  P = 0.488 | - | - | - | - | - | - | - | - |
| G × D | F _3,41_ = 1.01  P = 0.397 | F _3,41_ = 1.99  P = 0.130 | F _3.36_ = 0.57  P = 0.637 | F _3.36_ = 4.03  **P = 0.014** | - | - | - | - | - | - | - | - |
| I × G × D | F _3,41_ = 1.52  P = 0.222 | F _3,41_ = 1.08  P = 0.364 | F _3,36_ = 0.41  P = 0.744 | F _3,36_ = 0.41  P = 0.741 | - | - | - | - | - | - | - | - |
| Block | λ^2^ _1_ = 0  P = 1 | λ^2^ _1_ = -1.4 ^e-14^  P = 1 | λ^2^ _1_ = -3.5 ^e-15^  P = 1 | λ^2^ _1_ = 0.00  P = 0.926 | λ^2^ _1_ = -7.1 ^e-15^  P = 1 | λ^2^ _1_ = 1.26  P = 0.25 | λ^2^ _1_ = 0.33  P = 0.564 | λ^2^ _1_ = 1  P = 0.129 | - | - | - | - |

**Table S4.** ANOVA tests in LMMs for the effects of irrigation (I), genotype (G), developmental stages (D) and their interactions on bulk soil, rhizosphere, leaf, root, seed epiphytes and endophytes associated bacterial communities.

**Table S5.** ANOVA tests in LMMs for the effects of irrigation (I), genotype (G), developmental stages (D) and their interactions on bulk soil, rhizosphere, leaf, root, seed epiphytes and endophytes associated fungal communities.

| Fungal communities | | | | | | | | | | | | |
| --- | --- | --- | --- | --- | --- | --- | --- | --- | --- | --- | --- | --- |
|  | Bulk soil | | | | Rhizosphere | | | | Root | | | |
|  | With WSH | | Without WSH | | With WSH | | Without WSH | | With WSH | | Without WSH | |
|  | PCo1 | PCo2 | PCo1 | PCo2 | PCo1 | PCo2 | PCo1 | PCo2 | PCo1 | PCo2 | PCo1 | PCo2 |
| R^2^ | 0.47 | 0.58 | 0.62 | 0.76 | 0.51 | 0.59 | 0.45 | 0.71 | 0.69 | 0.41 | 0.78 | 0.44 |
| I | F _1,6_ = 0.00  P = 0.949 | F _1,6_ = 0.53  P = 0.492 | F _1,5_ = 13.0  **P = 0.011** | F _1,5_ = 17.1  **P = 0.006** | F _1,6_ = 0.75  P = 0.418 | F _1,6_ = 0.04  P = 0.842 | F _1,5_ = 1.24  P = 0.307 | F _1,5_ = 92.4  **P = 0.000** | F _1,5_ = 2.40  P = 0.172 | F _1,5_ = 0.48  P = 0.512 | F _1,5_ = 0.08  P = 0.775 | F _1,5_ = 0.48  P = 0.511 |
| G | F _3,66_ = 0.43  P = 0.731 | F _3,66_ = 0.51  P = 0.673 | F _3,62_ = 0.04  P = 0.985 | F _3,62_ = 0.10  P = 0.955 | F _3,42_ = 0.72  P = 0.542 | F _3,42_ = 0.55  P = 0.648 | F _3,38_ = 1.66  P = 0.190 | F _3,38_ = 0.27  P = 0.843 | F _3,41_ = 0.77  P = 0.514 | F _3,41_ = 2.26  P = 0.095 | F _3,41_ = 1.81  P = 0.160 | F _3,41_ = 1.26  P = 0.298 |
| D | F _2,66_ = 15.1  **P = 0.000** | F _2,66_ = 7.37  **P = 0.001** | F _2,62_ = 3.53  **P = 0.035** | F _2,62_ = 8.56  **P = 0.000** | F _1,42_ = 9.11  **P = 0.004** | F _1,42_ = 4.42  **P = 0.041** | F _1,39_ = 0.71  P = 0.404 | F _1,40_ = 50.0  **P = 0.000** | F _1,41_ = 100  **P = 0.000** | F _1,41_ = 6.80  **P = 0.012** | F _1,41_ = 148  **P = 0.000** | F _1,41_ = 18.2  **P = 0.000** |
| I × G | F _3,66_ = 1.08  P = 0.360 | F _3,66_ = 0.68  P = 0.564 | F _3,62_ = 0.99  P = 0.402 | F _3,62_ = 1.31  P = 0.277 | F _3,42_ = 0.94  P = 0.427 | F _3,42_ = 0.23  P = 0.874 | F _3,38_ = 1.26  P = 0.299 | F _3,38_ = 2.34  P = 0.088 | F _3,41_ = 0.24  P = 0.862 | F _3,41_ = 1.36  P = 0.268 | F _3,41_ = 1.50  P = 0.229 | F _3,41_ = 0.07  P = 0.973 |
| I × D | F _2,66_ = 1.12  P = 0.330 | F _2,66_ = 0.02  P = 0.977 | F _2,62_ = 2.47  P = 0.092 | F _2,62_ = 2.15  P = 0.124 | F _1,42_ = 2.80  P = 0.101 | F _1,42_ = 1.90  P = 0.175 | F _1,39_ = 3.89  P = 0.055 | F _1,41_ = 2.46  P = 0.124 | F _1,41_ = 1.86  P = 0.179 | F _1,41_ = 5.01  **P = 0.030** | F _1,41_ = 37.6  **P = 0.000** | F _1,41_ = 11.0  **P = 0.001** |
| G × D | F _6,66_ = 1.20  P = 0.314 | F _6,66_ = 1.04  P = 0.407 | F _6,62_ = 1.86  P = 0.101 | F _6,62_ = 0.43  P = 0.850 | F _3,42_ = 0.19  P = 0.898 | F _3,42_ = 0.93  P = 0.431 | F _3,38_ = 1.25  P = 0.304 | F _3,38_ = 0.05  P = 0.981 | F _3,41_ = 2.06  P = 0.120 | F _3,41_ = 2.62  P = 0.063 | F _3,41_ = 0.89  P = 0.451 | F _3,41_ = 0.40  P = 0.749 |
| I × G × D | F _6,66_ = 0.75  P = 0.610 | F _6,66_ = 0.24  P = 0.960 | F _6,62_ = 0.38  P = 0.884 | F _6,62_ = 0.87  P = 0.519 | F _3,42_ = 0.22  P = 0.880 | F _3,42_ = 1.69  P = 0.181 | F _3,38_ = 0.45  P = 0.717 | F _3,38_ = 0.55  P = 0.645 | F _3,41_ = 0.37  P = 0.769 | F _3,41_ = 0.61  P = 0.611 | F _3,41_ = 3.17  **P = 0.035** | F _3,41_ = 1.68  P = 0.186 |
| Block | λ^2^ _1_ = 12.2  **P = 0.000** | λ^2^ _1_ = 35.5  **P = 2.5 ^e-9^** | λ^2^ _1_ = 13.2  **P = 0.002** | λ^2^ _1_ = 24.5  **P = 7.4 ^e-7^** | λ^2^ _1_ = 24.5  **P = 0.002** | λ^2^ _1_ = 23.2  **P =** **1.4 ^e-6^** | λ^2^ _1_ = 6.82  **P =** **0.009** | λ^2^ _1_ = -2.8 ^e-14^  P = 1 | λ^2^ _1_ = 3.47  P = 0.062 | λ^2^ _1_ = 1.93  P = 0.164 | λ^2^ _1_ = 1.4 ^e-14^  P = 1 | λ^2^ _1_ = 0.73  P = 0.390 |
|  | Leaf | | | | Seed Epiphytes | | | | Seed Endophytes | | | |
|  | With WSH | | Without WSH | | With WSH | | Without WSH | | With WSH | | Without WSH | |
|  | PCo1 | PCo2 | PCo1 | PCo2 | PCo1 | PCo2 | PCo1 | PCo2 | PCo1 | PCo2 | PCo1 | PCo2 |
| R^2^ | 0.37 | 0.52 | 0.62 | 0.73 | 0.28 | 0.35 | 0.60 | 0.37 | 0.25 | 0.55 | 0.24 | 0.46 |
| I | F _1,5_ = 4.08  P = 0.091 | F _1,5_ = 0.62  P = 0.461 | F _1,4_ = 9.99  **P = 0.025** | F _1,5_ = 16.1  **P = 0.009** | F _1,6_ = 0.01  P = 0.923 | F _1,6_ = 1.40  P = 0.280 | F _1,6_ = 0.14  P = 0.719 | F _1,6_ = 2.43  P = 0.169 | F _1,6_ = 0.00  P = 0.941 | F _1,5_ = 0.13  P = 0.728 | F _1,5_ = 0.17  P = 0.678 | F _1,5_ = 2.31  P = 0.179 |
| G | F _3,37_ = 3.45  **P = 0.025** | F _3,37_ = 1.20  P = 0.320 | F _3,28_ = 1.49  P = 0.235 | F _3,28_ = 3.94  **P = 0.017** | F _3,18_ = 1.37  P = 0.283 | F _3,18_ = 3.34  **P = 0.042** | F _3,18_ = 6.71  **P = 0.003** | F _3,18_ = 3.99  **P = 0.024** | F _3,18_ = 1.82  P = 0.199 | F _3,16_ = 3.34  P = 0.058 | F _3,16_ = 0.38  P = 0.764 | F _3,16_ = 3.09  P = 0.055 |
| D | F _1,38_ = 0.00  P = 0.985 | F _1,38_ = 30.5  **P = 0.000** | F _1,29_ = 38.6  **P = 0.000** | F _1,29_ = 51.0  **P = 0.000** | - | - | - | - | - | - | - | - |
| I × G | F _3,37_ = 1.53  P = 0.220 | F _3,37_ = 3.38  **P = 0.027** | F _3,29_ = 1.43  P = 0.251 | F _3,28_ = 0.39  P = 0.754 | F _3,18_ = 2.54  P = 0.088 | F _3,18_ = 1.10  P = 0.373 | F _3,18_ = 2.00  P = 0.149 | F _3,18_ = 0.74  P = 0.537 | F _3,18_ = 0.43  P = 0.732 | F _3,16_ = 4.19  **P = 0.031** | F _3,16_ = 0.45  P = 0.716 | F _3,16_ = 1.49  P = 0.253 |
| I × D | F _1,38_ = 1.91  P = 0.174 | F _1,38_ = 1.40  P = 0.243 | F _1,29_ = 3.36  P = 0.076 | F _1,29_ = 7.50  **P = 0.010** | - | - | - | - | - | - | - | - |
| G × D | F _3,37_ = 1.70  P = 0.182 | F _3,37_ = 0.22  P = 0.877 | F _3,29_ = 6.24  **P = 0.002** | F _3,29_ = 3.17  **P = 0.038** | - | - | - | - | - | - | - | - |
| I × G × D | F _3,37_ = 1.62  P = 0.199 | F _3,37_ = 4.82  **P = 0.006** | F _3,29_ = 0.34  P = 0.790 | F _3,29_ = 1.18  P = 0.332 | - | - | - | - | - | - | - | - |
| Block | λ^2^ _1_ = 7.1 ^e-15^  P = 1 | λ^2^ _1_ = 0.02  P = 0.881 | λ^2^ _1_ = 7.1 ^e-15^  P = 1 | λ^2^ _1_ = 0.13  P = 0.715 | λ^2^ _1_ = 0.00  P = 0.949 | λ^2^ _1_ = 0.11  P = 0.734 | λ^2^ _1_ = 4.23  **P = 0.039** | λ^2^ _1_ = 0.03  P = 0.848 | λ^2^ _1_ = 0.0  P = 1 | λ^2^ _1_ = -3.5 ^e-15^  P = 1 | λ^2^ _1_ = 0.56  P = 0.453 | λ^2^ _1_ = 0.90  P = 0.340 |

| Bulk soil | | | | | | | | | |
| --- | --- | --- | --- | --- | --- | --- | --- | --- | --- |
| With WSH | | | | | | | | | |
|  | Acidobacteria | Actinobacteria | Bacteroidetes | Firmicutes | Gemmatimonadetes | Alpha-  proteobacteria | Beta-  proteobacteria | Delta -  proteobacteria | Gamma-  proteobacteria |
| R^2^ | 0.80 | 0.67 | 0.54 | 0.63 | 0.75 | 0.72 | 0.34 | 0.52 | 0.37 |
| I | F _1,6_ = 0.02  P = 0.875 | F _1,6_ = 0.01  P = 0.905 | F _1,6_ = 4.87  P = 0.069 | F _1,6_ = 0.00  P = 0.988 | F _1,6_ = 0.01  P = 0.893 | F _1,6_ = 3.59  P = 0.106 | F _1,6_ = 3.34  P = 0.117 | F _1,6_ = 0.02  P = 0.871 | F _1,6_ = 2.12  P = 0.194 |
| G | F _3,66_ = 2.61  P = 0.058 | F _3,66_ = 0.34  P = 0.793 | F _3,66_ = 0.43  P = 0.725 | F _3,66_ = 1.78  P = 0.158 | F _3,66_ = 0.05  P = 0.981 | F _3,66_ = 0.31  P = 0.814 | F _3,66_ = 1.10  P = 0.353 | F _3,66_ = 0.73  P = 0.532 | F _3,66_ = 0.58  P = 0.625 |
| D | F _2,66_ = 13.5  **P = 0.000** | F _2,66_ = 5.8  **P = 0.004** | F _2,66_ = 32.9  **P = 0.000** | F _2,66_ = 38.1  **P = 0.000** | F _2,66_ = 36.7  **P = 0.000** | F _2,66_ = 54.0  **P = 0.000** | F _2,66_ = 5.55  **P = 0.005** | F _2,66_ = 14.1  **P = 0.000** | F _2,66_ = 5.29  **P = 0.007** |
| I × G | F _3,66_ = 0.43  P = 0.731 | F _3,66_ = 1.05  P = 0.372 | F _3,66_ = 1.04  P = 0.379 | F _3,66_ = 1.50  P = 0.220 | F _3,66_ = 0.07  P = 0.975 | F _3,66_ = 1.02  P = 0.388 | F _3,66_ = 1.10  P = 0.354 | F _3,66_ = 0.26  P = 0.851 | F _3,66_ = 1.02  P = 0.387 |
| I × D | F _2,66_ = 4.63  **P = 0.013** | F _2,66_ = 9.41  **P = 0.000** | F _2,66_ = 2.55  P = 0.085 | F _2,66_ = 1.88  P = 0.159 | F _2,66_ = 9.45  **P = 0.000** | F _2,66_ = 32.1  **P = 0.000** | F _2,66_ = 1.01  P = 0.369 | F _2,66_ = 0.54  P = 0.583 | F _2,66_ = 1.48  P = 0.234 |
| G × D | F _6,66_ = 0.51  P = 0.794 | F _6,66_ = 2.35  **P = 0.039** | F _6,66_ = 0.76  P = 0.600 | F _6,66_ = 0.64  P = 0.696 | F _6,66_ = 0.89  P = 0.506 | F _6,66_ = 0.57  P = 0.751 | F _6,66_ = 0.93  P = 0.472 | F _6,66_ = 2.08  P = 0.066 | F _6,66_ = 0.59  P = 0.731 |
| I × G × D | F _6,66_ = 0.39  P = 0.879 | F _6,66_ = 1.70  P = 0.134 | F _6,66_ = 0.82  P = 0.556 | F _6,66_ = 1.24  P = 0.295 | F _6,66_ = 1.59  P = 0.161 | F _6,66_ = 0.68  P = 0.663 | F _6,66_ = 2.26  **P = 0.047** | F _6,66_ = 3.17  **P = 0.008** | F _6,66_ = 0.77  P = 0.588 |
| Block | λ^2^ _1_ = 83.6  **P = 2.2 ^e-15^** | λ^2^ _1_ = 45.4  **P = 1.5 ^e-11^** | λ^2^ _1_ = 3.94  **P = 0.047** | λ^2^ _1_ = 22.2  **P = 2.4 ^e-11^** | λ^2^ _1_ = 56.8  **P = 4.6 ^e-14^** | λ^2^ _1_ = 13.7  **P = 0.000** | λ^2^ _1_ = 0.56  P = 0.452 | λ^2^ _1_ = 14.0  **P = 0.000** | λ^2^ _1_ = 6.61  **P = 0.010** |
|  |  |  |  |  |  |  |  |  |  |
| Without WSH | | | | | | | | | |
|  | Acidobacteria | Actinobacteria | Bacteroidetes | Firmicutes | Gemmatimonadetes | Alpha-  proteobacteria | Beta-  proteobacteria | Delta -  proteobacteria | Gamma-  proteobacteria |
| R^2^ | 0.66 | 0.39 | 0.27 | 0.64 | 0.44 | 0.54 | 0.56 | 0.34 | 0.43 |
| I | F _1,5_ = 0.00  P = 0.938 | F _1,5_ = 0.16  P = 0.696 | F _1,5_ = 10.0  **P = 0.020** | F _1,5_ = 0.28  P = 0.614 | F _1,5_ = 4.35  P = 0.082 | F _1,5_ = 2.97  P = 0.135 | F _1,5_ = 0.21  P = 0.658 | F _1,5_ = 0.00  P = 0.939 | F _1,5_ = 4.51  P = 0.078 |
| G | F _3,62_ = 0.46  P = 0.705 | F _3,62_ = 1.79  P = 0.156 | F _3,62_ = 0.29  P = 0.828 | F _3,62_ = 0.5  P = 0.683 | F _3,62_ = 0.53  P = 0.661 | F _3,62_ = 0.01  P = 0.997 | F _3,62_ = 0.47  P = 0.704 | F _3,62_ = 0.34  P = 0.795 | F _3,62_ = 0.38  P = 0.761 |
| D | F _2,62_ = 3.92  **P = 0.024** | F _2,62_ = 5.95  **P = 0.004** | F _2,62_ = 2.42  P = 0.096 | F _2,62_ = 1.88  P = 0.160 | F _2,62_ = 9.48  **P = 0.000** | F _2,62_ = 11.9  **P = 0.000** | F _2,62_ = 21.7  **P = 0.000** | F _2,62_ = 12.0  **P = 0.000** | F _2,62_ = 0.45  P = 0.636 |
| I × G | F _3,62_ = 0.44  P = 0.719 | F _3,62_ = 1.13  P = 0.343 | F _3,62_ = 0.25  P = 0.857 | F _3,62_ = 1.33  P = 0.271 | F _3,62_ = 0.30  P = 0.821 | F _3,62_ = 0.72  P = 0.539 | F _3,62_ = 0.781  P = 0.508 | F _3,62_ = 0.09  P = 0.961 | F _3,62_ = 0.25  P = 0.860 |
| I × D | F _2,62_ = 16.2  **P = 0.000** | F _2,62_ = 0.13  P = 0.870 | F _2,62_ = 2.11  P = 0.129 | F _2,62_ = 0.34  P = 0.712 | F _2,62_ = 1.37  P = 0.260 | F _2,62_ = 6.08  **P = 0.003** | F _2,62_ = 6.94  **P = 0.001** | F _2,62_ = 1.14  P = 0.323 | F _2,62_ = 3.01  P = 0.056 |
| G × D | F _6,62_ = 0.24  P = 0.960 | F _6,62_ = 0.18  P = 0.978 | F _6,62_ = 0.87  P = 0.520 | F _6,62_ = 0.40  P = 0.870 | F _6,62_ = 1.00  P = 0.432 | F _6,62_ = 0.37  P = 0.892 | F _6,62_ = 4.25  **P = 0.001** | F _6,62_ = 0.97  P = 0.452 | F _6,62_ = 1.46  P = 0.206 |
| I × G × D | F _6,62_ = 1.94  P = 0.087 | F _6,62_ = 0.29  P = 0.935 | F _6,62_ = 0.77  P = 0.592 | F _6,62_ = 0.73  P = 0.622 | F _6,62_ = 0.66  P = 0.681 | F _6,62_ = 0.41  P = 0.868 | F _6,62_ = 1.97  P = 0.082 | F _6,62_ = 1.15  P = 0.343 | F _6,62_ = 1.29  P = 0.271 |
| Block | λ^2^ _1_ = 42.6  **P = 5.5 ^e-11^** | λ^2^ _1_ = 11.6  **P = 0.000** | λ^2^ _1_ = 0.01  P = 0.891 | λ^2^ _1_ = 46.4  **P = 9.5 ^e-12^** | λ^2^ _1_ = 46.4  **P = 9.5 ^e-12^** | λ^2^ _1_ = 16.2  **P = 5.9 ^e-5^** | λ^2^ _1_ = 3.43  P = 0.063 | λ^2^ _1_ = 0.62  P = 0.427 | λ^2^ _1_ = 8.44  **P = 0.003** |

**Table S6.** ANOVA tests in LMMs for the effects of irrigation (I), genotype (G), developmental stages (D) and their interactions on relative abundance of different bacterial phyla/classes associated with bulk soil, rhizosphere, leaf, root, seed epiphytes and endophytes.

| Rhizosphere | | | | | | | | | |
| --- | --- | --- | --- | --- | --- | --- | --- | --- | --- |
| With WSH | | | | | | | | | |
|  | Acidobacteria | Actinobacteria | Bacteroidetes | Firmicutes | Gemmatimonadetes | Alpha-  proteobacteria | Beta-  proteobacteria | Delta -  proteobacteria | Gamma-  proteobacteria |
| R^2^ | 0.70 | 0.73 | 0.51 | 0.54 | 0.72 | 0.90 | 0.60 | 0.44 | 0.29 |
| I | F _1,5_ = 0.03  P = 0.851 | F _1,5_ = 4.13  P = 0.088 | F _1,5_ = 0.42  P = 0.540 | F _1,5_ = 0.09  P = 0.768 | F _1,5_ = 1.58  P = 0.255 | F _1,5_ = 39.0  **P = 0.000** | F _1,5_ = 1.92  P = 0.215 | F _1,5_ = 1.04  P = 0.345 | F _1,5_ = 0.85  P = 0.391 |
| G | F _3,41_ = 1.11  P = 0.355 | F _3,41_ = 0.55  P = 0.650 | F _3,41_ = 1.42  P = 0.249 | F _3,41_ = 0.22  P = 0.881 | F _3,41_ = 0.48  P = 0.697 | F _3,41_ = 0.45  P = 0.716 | F _3,41_ = 2.10  P = 0.114 | F _3,41_ = 0.89  P = 0.451 | F _3,41_ = 0.56  P = 0.638 |
| D | F _1,41_ = 26.0  **P = 0.000** | F _1,41_ = 26.7  **P = 0.000** | F _1,41_ = 4.10  **P = 0.049** | F _1,41_ = 15.7  **P = 0.000** | F _1,41_ = 2.38  P = 0.130 | F _1,41_ = 129  **P = 0.000** | F _1,41_ = 52.4  **P = 0.000** | F _1,41_ = 13.3  **P = 0.000** | F _1,41_ = 1.67  P = 0.202 |
| I × G | F _3,41_ = 1.57  P = 0.210 | F _3,41_ = 1.07  P = 0.372 | F _3,41_ = 4.14  **P = 0.011** | F _3,41_ = 0.63  P = 0.599 | F _3,41_ = 0.23  P = 0.868 | F _3,41_ = 2.98  **P = 0.041** | F _3,41_ = 0.42  P = 0.739 | F _3,41_ = 1.32  P = 0.279 | F _3,41_ = 1.33  P = 0.274 |
| I × D | F _1,41_ = 3.68  P = 0.061 | F _1,41_ = 5.01  **P = 0.030** | F _1,41_ = 1.85  P = 0.180 | F _1,41_ = 13.3  **P = 0.000** | F _1,41_ = 2.77  P = 0.103 | F _1,41_ = 167  **P = 4.5 ^e-16^** | F _1,41_ = 4.86  **P = 0.032** | F _1,41_ = 0.04  P = 0.828 | F _1,41_ = 0.65  P = 0.422 |
| G × D | F _3,41_ = 1.02  P = 0.393 | F _3,41_ = 5.62  **P = 0.002** | F _3,41_ = 0.90  P = 0.448 | F _3,41_ = 0.08  P = 0.965 | F _3,41_ = 2.03  P = 0.123 | F _3,41_ = 0.10  P = 0.956 | F _3,41_ = 2.77  P = 0.053 | F _3,41_ = 0.96  P = 0.418 | F _3,41_ = 1.02  P = 0.393 |
| I × G × D | F _3,41_ = 0.409  P = 0.746 | F _3,41_ = 1.04  P = 0.384 | F _3,41_ = 1.32  P = 0.280 | F _3,41_ = 0.73  P = 0.540 | F _3,41_ = 0.83  P = 0.480 | F _3,41_ = 1.06  P = 0.375 | F _3,41_ = 0.20  P = 0.892 | F _3,41_ = 1.13  P = 0.344 | F _3,41_ = 2.35  P = 0.086 |
| Block | λ^2^ _1_ = 29.0  **P = 6.9 ^e-8^** | λ^2^ _1_ = 21.4  **P = 3.5 ^e-6^** | λ^2^ _1_ = 10.3  **P = 0.012** | λ^2^ _1_ = 11.7  **P = 0.000** | λ^2^ _1_ = 33.6  **P = 6.6 ^e-9^** | λ^2^ _1_ = 11.7  **P = 0.000** | λ^2^ _1_ = 2.05  P = 0.151 | λ^2^ _1_ = 5.17  **P = 0.022** | λ^2^ _1_ = 0.72  P = 0.394 |
|  |  |  |  |  |  |  |  |  |  |
| Without WSH | | | | | | | | | |
|  | Acidobacteria | Actinobacteria | Bacteroidetes | Firmicutes | Gemmatimonadetes | Alpha-  proteobacteria | Beta-  proteobacteria | Delta -  proteobacteria | Gamma-  proteobacteria |
| R^2^ | 0.64 | 0.38 | 0.29 | 0.67 | 0.46 | 0.80 | 0.70 | 0.32 | 0.61 |
| I | F _1,5_ = 0.60  P = 0.465 | F _1,5_ = 0.26  P = 0.625 | F _1,5_ = 1.49  P = 0.270 | F _1,5_ = 0.51  P = 0.498 | F _1,5_ = 1.74  P = 0.235 | F _1,5_ = 47.4  **P = 0.000** | F _1,5_ = 20.4  **P = 0.004** | F _1,5_ = 0.45  P = 0.525 | F _1,5_ = 2.7  P = 0.147 |
| G | F _3,37_ = 1.16  P = 0.334 | F _3,37_ = 1.87  P = 0.150 | F _3,37_ = 1.05  P = 0.378 | F _3,37_ = 0.49  P = 0.686 | F _3,37_ = 1.25  P = 0.303 | F _3,37_ = 3.2  **P = 0.033** | F _3,37_ = 0.49  P = 0.688 | F _3,37_ = 0.80  P = 0.497 | F _3,37_ = 5.8  **P = 0.002** |
| D | F _1,38_ = 44.2  **P = 0.000** | F _1,38_ = 0.00  P = 0.937 | F _1,38_ = 4.01  **P = 0.052** | F _1,38_ = 72.4  **P = 0.000** | F _1,38_ = 0.03  P = 0.860 | F _1,38_ = 53.2  **P = 0.000** | F _1,38_ = 59.0  **P = 0.000** | F _1,38_ = 13.0  **P = 0.000** | F _1,38_ = 6.9  **P = 0.011** |
| I × G | F _3,37_ = 0.41  P = 0.743 | F _3,37_ = 1.17  P = 0.331 | F _3,37_ = 1.03  P = 0.388 | F _3,37_ = 0.25  P = 0.856 | F _3,37_ = 0.46  P = 0.711 | F _3,37_ = 1.46  P = 0.239 | F _3,37_ = 0.71  P = 0.549 | F _3,37_ = 0.28  P = 0.835 | F _3,37_ = 3.01  **P = 0.041** |
| I × D | F _1,38_ = 3.15  P = 0.083 | F _1,38_ = 0.06  P = 0.803 | F _1,38_ = 1.65  P = 0.205 | F _1,38_ = 0.01  P = 0.893 | F _1,38_ = 9.30  **P = 0.004** | F _1,38_ = 1.34  P = 0.253 | F _1,38_ = 2.44  P = 0.126 | F _1,38_ = 7.15  **P = 0.010** | F _1,38_ = 0.34  P = 0.561 |
| G × D | F _3,37_ = 0.47  P = 0.702 | F _3,37_ = 0.29  P = 0.828 | F _3,37_ = 0.42  P = 0.733 | F _3,37_ = 0.20  P = 0.893 | F _3,37_ = 0.87  P = 0.461 | F _3,37_ = 1.29  P = 0.290 | F _3,37_ = 0.69  P = 0.560 | F _3,37_ = 0.80  P = 0.499 | F _3,37_ = 0.19  P = 0.899 |
| I × G × D | F _3,37_ = 1.21  P = 0.317 | F _3,37_ = 0.47  P = 0.700 | F _3,37_ = 0.85  P = 0.471 | F _3,37_ = 1.04  P = 0.382 | F _3,37_ = 1.58  P = 0.210 | F _3,37_ = 1.60  P = 0.204 | F _3,37_ = 1.70  P = 0.182 | F _3,37_ = 0.06  P = 0.975 | F _3,37_ = 1.20  P = 0.320 |
| Block | λ^2^ _1_ = 12.3  **P = 0.000** | λ^2^ _1_ = 6.54  **P = 0.010** | λ^2^ _1_ = 0.74  P = 0.387 | λ^2^ _1_ = 10.9  **P = 0.000** | λ^2^ _1_ = 5.27  **P = 0.021** | λ^2^ _1_ = 2.82  P = 0.092 | λ^2^ _1_ = 3.05  P = 0.080 | λ^2^ _1_ = 5.6 ^e-14^  P = 1 | λ^2^ _1_ = 10.7  **P = 0.001** |

| Root | | | | | | | | | |
| --- | --- | --- | --- | --- | --- | --- | --- | --- | --- |
| With WSH | | | | | | | | | |
|  | Acidobacteria | Actinobacteria | Bacteroidetes | Firmicutes | Gemmatimonadetes | Alpha-  proteobacteria | Beta-  proteobacteria | Delta -  proteobacteria | Gamma-  proteobacteria |
| R^2^ | 0.44 | 0.32 | 0.41 | 0.41 | 0.44 | 0.58 | 0.62 | 0.47 | 0.38 |
| I | F _1,6_ = 0.00  P = 0.943 | F _1,6_ = 0.00  P = 0.990 | F _1,6_ = 2.87  P = 0.140 | F _1,6_ = 0.09  P = 0.763 | F _1,6_ = 0.95  P = 0.366 | F _1,6_ = 2.25  P = 0.184 | F _1,6_ = 16.2  **P = 0.006** | F _1,6_ = 0.04  P = 0.847 | F _1,6_ = 0.10  P = 0.752 |
| G | F _3,42_ = 0.84  P = 0.476 | F _3,42_ = 0.30  P = 0.822 | F _3,42_ = 0.98  P = 0.408 | F _3,42_ = 3.85  **P = 0.015** | F _3,42_ = 0.61  P = 0.608 | F _3,42_ = 0.87  P = 0.459 | F _3,42_ = 0.95  P = 0.420 | F _3,42_ = 6.06  **P = 0.001** | F _3,42_ = 1.00  P = 0.401 |
| D | F _1,42_ = 0.39  P = 0.532 | F _1,42_ = 6.80  **P = 0.012** | F _1,42_ = 1.39  P = 0.245 | F _1,42_ = 13.6  **P = 0.000** | F _1,42_ = 3.69  P = 0.0612 | F _1,42_ = 16.0  **P = 0.000** | F _1,42_ = 62.7  **P = 0.000** | F _1,42_ = 2.80  P = 0.101 | F _1,42_ = 25.8  **P = 0.000** |
| I × G | F _3,42_ = 1.02  P = 0.389 | F _3,42_ = 0.61  P = 0.610 | F _3,42_ = 1.53  P = 0.219 | F _3,42_ = 1.72  P = 0.175 | F _3,42_ = 1.10  P = 0.359 | F _3,42_ = 1.25  P = 0.300 | F _3,42_ = 0.96  P = 0.420 | F _3,42_ = 2.46  P = 0.075 | F _3,42_ = 1.04  P = 0.383 |
| I × D | F _1,42_ = 1.90  P = 0.174 | F _1,42_ = 2.86  P = 0.098 | F _1,42_ = 0.07  P = 0.785 | F _1,42_ = 0.00  P = 0.958 | F _1,42_ = 0.56  P = 0.455 | F _1,42_ = 13.0  **P = 0.000** | F _1,42_ = 16.7  **P = 0.000** | F _1,42_ = 4.92  **P = 0.031** | F _1,42_ = 0.00  P = 0.989 |
| G × D | F _3,42_ = 0.31  P = 0.816 | F _3,42_ = 0.64  P = 0.592 | F _3,42_ = 0.75  P = 0.525 | F _3,42_ = 2.23  P = 0.098 | F _3,42_ = 0.63  P = 0.594 | F _3,42_ = 1.96  P = 0.133 | F _3,42_ = 0.09  P = 0.962 | F _3,42_ = 1.48  P = 0.232 | F _3,42_ = 0.80  P = 0.497 |
| I × G × D | F _3,42_ = 0.78  P = 0.511 | F _3,42_ = 1.75  P = 0.169 | F _3,42_ = 1.14  P = 0.342 | F _3,42_ = 1.28  P = 0.292 | F _3,42_ = 0.47  P = 0.701 | F _3,42_ = 1.10  P = 0.355 | F _3,42_ = 0.27  P = 0.846 | F _3,42_ = 1.77  P = 0.165 | F _3,42_ = 0.52  P = 0.670 |
| Block | λ^2^ _1_ = 11.9  **P = 0.000** | λ^2^ _1_ = 2.10  **P = 0.146** | λ^2^ _1_ = 5.12  **P = 0.023** | λ^2^ _1_ = 0.38  P = 0.534 | λ^2^ _1_ = 9.66  **P = 0.001** | λ^2^ _1_ = 9.71  **P = 0.001** | λ^2^ _1_ = 0  **P = 1** | λ^2^ _1_ = 3.42  P = 0.064 | λ^2^ _1_ = 0.18  P = 0.655 |
|  |  |  |  |  |  |  |  |  |  |
| Without WSH | | | | | | | | | |
|  | Acidobacteria | Actinobacteria | Bacteroidetes | Firmicutes | Gemmatimonadetes | Alpha-  proteobacteria | Beta-  proteobacteria | Delta -  proteobacteria | Gamma-  proteobacteria |
| R^2^ | 0.26 | 0.26 | 0.49 | 0.29 | 0.36 | 0.26 | 0.74 | 0.49 | 0.38 |
| I | F _1,5_ = 2.46  P = 0.169 | F _1,5_ = 0.12  P = 0.736 | F _1,5_ = 4.77  P = 0.073 | F _1,5_ = 1.96  P = 0.213 | F _1,5_ = 1.94  P = 0.214 | F _1,5_ = 2.03  P = 0.206 | F _1,5_ = 5.21  P = 0.063 | F _1,5_ = 1.70  P = 0.240 | F _1,5_ = 0.25  P = 0.631 |
| G | F _3,38_ = 0.34  P = 0.790 | F _3,38_ = 0.74  P = 0.529 | F _3,38_ = 0.40  P = 0.749 | F _3,38_ = 1.29  P = 0.288 | F _3,38_ = 0.96  P = 0.417 | F _3,38_ = 0.15  P = 0.929 | F _3,38_ = 0.89  P = 0.454 | F _3,38_ = 1.68  P = 0.187 | F _3,38_ = 0.46  P = 0.706 |
| D | F _1,40_ = 0.34  P = 0.561 | F _1,40_ = 4.78  **P = 0.034** | F _1,40_ = 23.7  **P = 0.000** | F _1,40_ = 7.13  **P = 0.010** | F _1,40_ = 0.93  P = 0.340 | F _1,40_ = 3.39  P = 0.072 | F _1,40_ = 110  **P = 0.000** | F _1,40_ = 7.61  **P = 0.008** | F _1,40_ = 15.8  **P = 0.000** |
| I × G | F _3,38_ = 1.48  P = 0.234 | F _3,38_ = 2.11  P = 0.113 | F _3,38_ = 0.60  P = 0.618 | F _3,38_ = 1.15  P = 0.338 | F _3,38_ = 2.22  P = 0.100 | F _3,38_ = 1.24  P = 0.307 | F _3,38_ = 1.03  P = 0.386 | F _3,38_ = 0.87  P = 0.460 | F _3,38_ = 2.30  P = 0.092 |
| I × D | F _1,40_ = 0.14  P = 0.709 | F _1,40_ = 0.26  P = 0.607 | F _1,40_ = 10.5  **P = 0.002** | F _1,40_ = 1.01  P = 0.320 | F _1,40_ = 4.51  **P = 0.039** | F _1,40_ = 6.34  **P = 0.015** | F _1,40_ = 13.2  **P = 0.000** | F _1,40_ = 25.4  **P = 0.000** | F _1,40_ = 0.29  P = 0.587 |
| G × D | F _3,38_ = 0.14  P = 0.933 | F _3,38_ = 0.30  P = 0.823 | F _3,38_ = 0.22  P = 0.880 | F _3,38_ = 1.55  P = 0.214 | F _3,38_ = 0.47  P = 0.701 | F _3,38_ = 0.49  P = 0.686 | F _3,38_ = 0.40  P = 0.748 | F _3,38_ = 0.55  P = 0.647 | F _3,38_ = 0.54  P = 0.653 |
| I × G × D | F _3,38_ = 0.52  P = 0.667 | F _3,38_ = 1.01  P = 0.395 | F _3,38_ = 0.26  P = 0.853 | F _3,38_ = 1.08  P = 0.367 | F _3,38_ = 2.78  **P = 0.053** | F _3,38_ = 0.65  P = 0.586 | F _3,38_ = 1.01  P = 0.396 | F _3,38_ = 0.88  P = 0.458 | F _3,38_ = 1.38  P = 0.263 |
| Block | λ^2^ _1_ = 1.61  P = 0.204 | λ^2^ _1_ = 0.21  P = 0.642 | λ^2^ _1_ = 0.55  P = 0.456 | λ^2^ _1_ = -5.6 ^e-14^  P = 1 | λ^2^ _1_ = 0.166  P = 0.683 | λ^2^ _1_ = 0.00  P = 0.957 | λ^2^ _1_ = 4.52  P = 0.033 | λ^2^ _1_ = 0.73  P = 0.390 | λ^2^ _1_ = 0.64  P = 0.420 |

| Leaf | | | | | | | | | |
| --- | --- | --- | --- | --- | --- | --- | --- | --- | --- |
| With WSH | | | | | | | | | |
|  | Acidobacteria | Actinobacteria | Bacteroidetes | Firmicutes | Gemmatimonadetes | Alpha-  proteobacteria | Beta-  proteobacteria | Delta -  proteobacteria | Gamma-  proteobacteria |
| R^2^ | 0.18 | 0.34 | 0.49 | 0.18 | 0.37 | 0.33 | 0.26 | 0.25 | 0.36 |
| I | F _1,5_ = 0.00  P = 0.994 | F _1,5_ = 0.01  P = 0.897 | F _1,5_ = 0.08  P = 0.781 | F _1,5_ = 0.37  P = 0.564 | F _1,5_ = 2.14  P = 0.193 | F _1,5_ = 2.39  P = 0.173 | F _1,5_ = 0.02  P = 0.890 | F = 0.77  P = 0.413 | F = 0.36  P = 0.568 |
| G | F _3,40_ = 1.27  P = 0.294 | F _3,40_ = 3.38  **P = 0.027** | F _3,40_ = 2.52  P = 0.071 | F _3,40_ = 0.38  P = 0.761 | F _3,40_ = 1.24  P = 0.305 | F _3,40_ = 1.08  P = 0.366 | F _3,40_ = 1.57  P = 0.210 | F = 0.86  P = 0.465 | F = 2.43  P = 0.079 |
| D | F _1,40_ = 0.51  P = 0.478 | F _1,40_ = 5.00  **P = 0.030** | F _1,40_ = 23.9  **P = 0.000** | F _1,40_ = 1.48  P = 0.229 | F _1,40_ = 9.55  **P = 0.003** | F _1,40_ = 6.79  **P = 0.012** | F _1,40_ = 3.81  P = 0.057 | F = 0.31  P = 0.575 | F = 16.1  **P = 0.000** |
| I × G | F _3,40_ = 0.19  P = 0.896 | F _3,40_ = 2.16  P = 0.106 | F _3,40_ = 0.96  P = 0.417 | F _3,40_ = 0.76  P = 0.518 | F _3,40_ = 3.16  **P = 0.034** | F _3,40_ = 3.07  **P = 0.038** | F _3,40_ = 0.35  P = 0.784 | F = 1.88  P = 0.148 | F = 0.46  P = 0.706 |
| I × D | F _1,40_ = 1.35  P = 0.251 | F _1,40_ = 1.24  P = 0.270 | F _1,40_ = 1.01  P = 0.319 | F _1,40_ = 1.29  P = 0.261 | F _1,40_ = 0.01  P = 0.917 | F _1,40_ = 0.12  P = 0.725 | F _1,40_ = 4.24  **P = 0.045** | F = 1.27  P = 0.265 | F = 0.20  P = 0.652 |
| G × D | F _3,40_ = 2.00  P = 0.128 | F _3,40_ = 2.01  P = 0.127 | F _3,40_ = 3.15  **P = 0.035** | F _3,40_ = 1.22  P = 0.312 | F _3,40_ = 0.132  P = 0.940 | F _3,40_ = 1.57  P = 0.210 | F _3,40_ = 0.20  P = 0.892 | F = 0.85  P = 0.470 | F = 2.46  P = 0.075 |
| I × G × D | F _3,40_ = 0.16  P = 0.916 | F _3,40_ = 0.32  P = 0.810 | F _3,40_ = 0.81  P = 0.495 | F _3,40_ = 1.19  P = 0.322 | F _3,40_ = 0.68  P = 0.569 | F _3,40_ = 1.10  P = 0.357 | F _3,40_ = 1.84  P = 0.154 | F = 2.25  P = 0.096 | F = 0.22  P = 0.875 |
| Block | λ^2^ _1_ = 0.03  P = 0.850 | λ^2^ _1_ = 0  P = 1 | λ^2^ _1_ = 2.42  P = 0.119 | λ^2^ _1_ = 0  P = 1 | λ^2^ _1_ = 0.84  P = 0.357 | λ^2^ _1_ = -2.8 ^e-14^  P = 1 | λ^2^ _1_ = 0.12  P = 0.718 | λ^2^ _1_ = 0  P = 1 | λ^2^ _1_ = -2.1 ^e-14^  P = 1 |
|  |  |  |  |  |  |  |  |  |  |
| Without WSH | | | | | | | | | |
|  | Acidobacteria | Actinobacteria | Bacteroidetes | Firmicutes | Gemmatimonadetes | Alpha-  proteobacteria | Beta-  proteobacteria | Delta -  proteobacteria | Gamma-  proteobacteria |
| R^2^ | 0.35 | 0.42 | 0.37 | 0.53 | 0.26 | 0.36 | 0.39 | 0.27 | 0.65 |
| I | F _1,5_ = 0.00  P = 0.993 | F _1,5_ = 1.16  P = 0.324 | F _1,5_ = 0.01  P = 0.916 | F _1,5_ = 0.50  P = 0.503 | F _1,5_ = 0.28  P = 0.616 | F _1,5_ = 0.03  P = 0.867 | F = 1.7  P = 0.240 | F = 0.07  P = 0.798 | F = 0.12  P = 0.734 |
| G | F _3,35_ = 1.22  P = 0.314 | F _3,35_ = 4.26  **P = 0.011** | F _3,35_ = 1.77  P = 0.170 | F _3,35_ = 4.72  **P = 0.007** | F _3,35_ = 1.36  P = 0.270 | F _3,35_ = 0.77  P = 0.514 | F = 0.31  P = 0.816 | F = 1.90  P = 0.146 | F = 5.76  **P = 0.002** |
| D | F _1,36_ = 9.34  **P = 0.004** | F _1,36_ = 4.19  **P = 0.047** | F _1,36_ = 12.0  **P = 0.001** | F _1,36_ = 14.6  **P = 0.000** | F _1,36_ = 5.40  **P = 0.025** | F _1,36_ = 13.2  **P = 0.000** | F = 2.10  P = 0.155 | F = 3.33  P = 0.075 | F = 47.7  **P = 0.000** |
| I × G | F _3,35_ = 1.00  P = 0.401 | F _3,35_ = 0.40  P = 0.748 | F _3,35_ = 1.02  P = 0.394 | F _3,35_ = 0.16  P = 0.918 | F _3,35_ = 1.28  P = 0.294 | F _3,35_ = 0.45  P = 0.713 | F = 1.68  P = 0.187 | F = 0.20  P = 0.895 | F = 0.70  P = 0.555 |
| I × D | F _1,37_ = 0.01  P = 0.905 | F _1,37_ = 0.50  P = 0.482 | F _1,37_ = 0.23  P = 0.631 | F _1,37_ = 1.80  P = 0.187 | F _1,37_ = 0.07  P = 0.781 | F _1,37_ = 4.54  **P = 0.039** | F = 2.66  P = 0.111 | F = 0.02  P = 0.871 | F = 2.50  P = 0.122 |
| G × D | F _3,35_ = 1.12  P = 0.350 | F _3,35_ = 1.36  P = 0.270 | F _3,35_ = 3.01  **P = 0.042** | F _3,35_ = 5.73  **P = 0.002** | F _3,35_ = 1.38  P = 0.263 | F _3,35_ = 1.70  P = 0.183 | F = 1.55  P = 0.216 | F = 1.81  P = 0.161 | F = 2.06  P = 0.122 |
| I × G × D | F _3,35_ = 0.89  P = 0.454 | F _3,35_ = 4.60  **P = 0.007** | F _3,35_ = 1.30  P = 0.289 | F _3,35_ = 2.47  P = 0.077 | F _3,35_ = 0.48  P = 0.691 | F _3,35_ = 0.66  P = 0.582 | F = 1.34  P = 0.274 | F = 0.50  P = 0.678 | F = 3.2  **P = 0.033** |
| Block | λ^2^ _1_ = 1.08  P = 0.297 | λ^2^ _1_ = 0.11  P = 0.735 | λ^2^ _1_ = -1.4 ^e-14^  P = 1 | λ^2^ _1_ = 0.70  P = 0.401 | λ^2^ _1_ = 2.8 ^e-14^  P = 1 | λ^2^ _1_ = 0.25  P = 0.612 | λ^2^ _1_ = 2.12  P = 0.145 | λ^2^ _1_ = 1  P = 0.550 | λ^2^ _1_ = 2.47  P = 0.115 |

| Seed Epiphytes | | | | | | | | | |
| --- | --- | --- | --- | --- | --- | --- | --- | --- | --- |
| With WSH | | | | | | | | | |
|  | Acidobacteria | Actinobacteria | Bacteroidetes | Firmicutes | Gemmatimonadetes | Alpha-  proteobacteria | Beta-  proteobacteria | Delta -  proteobacteria | Gamma-  proteobacteria |
| R^2^ | 0.35 | 0.26 | 0.36 | 0.28 | 0.39 | 0.48 | 0.37 | 0.11 | 0.33 |
| I | F _1,5_ = 2.89  P = 0.140 | F _1,5_ = 1.24  P = 0.307 | F _1,5_ = 0.33  P = 0.584 | F _1,5_ = 0.00  P = 0.991 | F _1,5_ = 6.96  **P = 0.039** | F _1,5_ = 0.18  P = 0.682 | F _1,5_ = 0.20  P = 0.664 | F _1,5_ = 0.81  P = 0.402 | F _1,5_ = 0.89  P = 0.381 |
| G | F _3,17_ = 0.61  P = 0.636 | F _3,17_ = 1.19  P = 0.339 | F _3,17_ = 2.48  P = 0.095 | F _3,17_ = 2.89  P = 0.065 | F _3,17_ = 3.04  P = 0.056 | F _3,17_ = 3.54  **P = 0.036** | F _3,17_ = 2.93  P = 0.062 | F _3,17_ = 0.87  P = 0.516 | F _3,17_ = 2.23  P = 0.121 |
| D | - | - | - | - | - | - | - | - | - |
| I × G | F _3,17_ = 0.40  P = 0.747 | F _3,17_ = 1.57  P = 0.232 | F _3,17_ = 1.55  P = 0.235 | F _3,17_ = 0.97  P = 0.425 | F _3,17_ = 0.97  P = 0.425 | F _3,17_ = 2.15  P = 0.130 | F _3,17_ = 1.50  P = 0.248 | F _3,17_ = 0.17  P = 0.915 | F _3,17_ = 1.91  P = 0.165 |
| I × D | - | - | - | - | - | - | - | - | - |
| G × D | - | - | - | - | - | - | - | - | - |
| I × G × D | - | - | - | - | - | - | - | - | - |
| Block | λ^2^ _1_ = 1.24  P = 0.264 | λ^2^ _1_ = 0.01  P = 0.897 | λ^2^ _1_ = 0.44  P = 0.505 | λ^2^ _1_ = **-1.4 ^e-14^**  P = 1 | λ^2^ _1_ = 5.6 ^e-14^  P = 1 | λ^2^ _1_ = 1.88  P = 0.170 | λ^2^ _1_ = 0.39  P = 0.532 | λ^2^ _1_ = -5.8 ^e-5^  P = 0.993 | λ^2^ _1_ = 0.02  P = 0.869 |
|  |  |  |  |  |  |  |  |  |  |
| Without WSH | | | | | | | | | |
|  | Acidobacteria | Actinobacteria | Bacteroidetes | Firmicutes | Gemmatimonadetes | Alpha-  proteobacteria | Beta-  proteobacteria | Delta -  proteobacteria | Gamma-  proteobacteria |
| R^2^ | 0.17 | 0.67 | 0.31 | 0.21 | 0.18 | 0.29 | 0.17 | 0.18 | 0.58 |
| I | F _1,5_ = 0.78  P = 0.409 | F _1,5_ = 5.05  P = 0.066 | F _1,5_ = 0.74  P = 0.434 | F _1,5_ = 0.02  P = 0.879 | F _1,5_ = 1.06  P = 0.330 | F _1,5_ = 0.55  P = 0.483 | F _1,5_ = 1.00  P = 0.354 | F _1,5_ = 1.03  P = 0.349 | F _1,5_ = 1.93  P = 0.213 |
| G | F _3,17_ = 0.80  P = 0.510 | F _3,17_ = 14.07  **P = 6.8 ^e-5^** | F _3,17_ = 1.43  P = 0.279 | F _3,17_ = 1.27  P = 0.314 | F _3,17_ = 0.94  P = 0.440 | F _3,17_ = 2.99  P = 0.059 | F _3,17_ = 0.94  P = 0.439 | F _3,17_ = 0.95  P = 0.436 | F _3,17_ = 8.55  **P = 0.001** |
| D | - | - | - | - | - | - | - | - | - |
| I × G | F _3,17_ = 1.01  P = 0.411 | F _3,17_ = 4.84  **P = 0.012** | F _3,17_ = 0.68  P = 0.571 | F _3,17_ = 0.98  P = 0.421 | F _3,17_ = 0.92  P = 0.451 | F _3,17_ = 0.39  P = 0.756 | F _3,17_ = 0.76  P = 0.530 | F _3,17_ = 0.92  P = 0.447 | F _3,17_ = 3.21  **P = 0.048** |
| I × D | - | - | - | - | - | - | - | - | - |
| G × D | - | - | - | - | - | - | - | - | - |
| I × G × D | - | - | - | - | - | - | - | - | - |
| Block | λ^2^ _1_ = 1  P = -2.8 ^e-14^ | λ^2^ _1_ = 0.01  P = 0.900 | λ^2^ _1_ = 0.80  P = 0.369 | λ^2^ _1_ = 0.03  P = 0.862 | λ^2^ _1_ = 0  P = 1 | λ^2^ _1_ = 0.07  P = 0.790 | λ^2^ _1_ = **-2.8 ^e-14^**  P = 1 | λ^2^ _1_ = 0  P = 1 | λ^2^ _1_ = 0.34  P = 0.555 |

**Table S7.** ANOVA tests for the effects of irrigation (I), genotype (G), developmental stages (D) and their interactions on relative abundance of different fungal phyla associated with bulk soil, rhizosphere, leaf, root, seed epiphytes and endophytes.

| Bulk soil | | | | | | |
| --- | --- | --- | --- | --- | --- | --- |
| Factor | **With WSH** | | | **Without WSH** | | |
|  | Ascomycota | Basidiomycota | Zygomycota | Ascomycota | Basidiomycota | Zygomycota |
| R^2^ | 0.33 | 0.38 | 0.30 | 0.31 | 0.24 | 0.25 |
| I | F _1,6_ = 16.3  **P = 0.006** | F _1,6_ = 0.00  P = 0.965 | F _1,6_ = 10.9  **P = 0.016** | F _1,5_ = 2.08  P = 0.199 | F _1,5_ = 0.14  P = 0.718 | F _1,5_ = 4.50  P = 0.079 |
| G | F _3,66_ = 0.89  P = 0.449 | F _3,66_ = 0.93  P = 0.430 | F _3,66_ = 0.49  P = 0.687 | F _3,61_ = 0.20  P = 0.893 | F _3,61_ = 0.87  P = 0.460 | F _3,61_ = 0.74  P = 0.528 |
| D | F _2,66_ = 1.54  P = 0.220 | F _2,66_ = 6.10  P = 0.003 | F _2,66_ = 4.84  **P = 0.010** | F _2,62_ = 0.44  P = 0.641 | F _2,62_ = 0.51  P = 0.599 | F _2,62_ = 0.47  P = 0.623 |
| I × G | F _3,66_ = 1.91  P = 0.135 | F _3,66_ = 0.42  P = 0.734 | F _3,66_ = 0.85  P = 0.468 | F _3,61_ = 0.37  P = 0.770 | F _3,61_ = 0.17  P = 0.910 | F _3,61_ = 0.20  P = 0.891 |
| I × D | F _2,66_ = 1.48  P = 0.234 | F _2,66_ = 1.09  P = 0.340 | F _2,66_ = 0.64  P = 0.528 | F _2,63_ = 1.96  P = 0.148 | F _2,63_ = 0.57  P = 0.564 | F _2,63_ = 7.74  **P = 0.000** |
| G × D | F _6,66_ = 0.96  P = 0.454 | F _6,66_ = 0.964  P = 0.455 | F _6,66_ = 1.64  P = 0.149 | F _6,61_ = 0.40  P = 0.873 | F _6,61_ = 0.84  P = 0.539 | F _6,61_ = 0.68  P = 0.664 |
| I × G × D | F _6,66_ = 0.352  P = 0.9061 | F _6,66_ = 0.27  P = 0.948 | F _6,66_ = 0.34  P = 0.910 | F _6,61_ = 1.15  P = 0.341 | F _6,61_ = 0.76  P = 0.600 | F _6,61_ = 0.41  P = 0.866 |
| Block | λ^2^ _1_ = 0.21  P = 0.645 | λ^2^ _1_ = 11.2  **P = 0.000** | λ^2^ _1_ = 0.42  P = 0.638 | λ^2^ _1_ = 4.98  **P = 0.025** | λ^2^ _1_ = 2.45  P = 0.116 | λ^2^ _1_ = 5.6 ^e-14^  P = 1 |
|  |  |  |  |  |  |  |
| Rhizosphere | | | | | | |
|  | **With WSH** | | | **Without WSH** | | |
|  | Ascomycota | Basidiomycota | Zygomycota | Ascomycota | Basidiomycota | Zygomycota |
| R^2^ | 0.40 | 0.47 | 0.36 | 0.42 | 0.44 | 0.36 |
| I | F _1,6_ = 1.27  P = 0.302 | F _1,5_ = 0.105  P = 0.756 | F _1,5_ = 8.95  **P = 0.024** | F _1,5_ = 5.89  **P = 0.053** | F _1,5_ = 0.75  P = 0.417 | F _1,5_ = 6.68  **P = 0.044** |
| G | F _3,41_ = 0.31  P = 0.811 | F _3,41_ = 1.20  P = 0.320 | F _3,41_ = 0.62  P = 0.600 | F _3,37_ = 0.25  P = 0.854 | F _3,37_ = 2.36  P = 0.087 | F _3,37_ = 0.47  P = 0.702 |
| D | F _1,41_ = 1.29  P = 0.260 | F _1,41_ = 12.7  **P = 0.000** | F _1,41_ = 15.6  **P = 0.000** | F _1,38_ = 6.17  **P = 0.017** | F _1,38_ = 0.12  P = 0.720 | F _1,38_ = 8.61  **P = 0.005** |
| I × G | F _3,41_ = 0.56  P = 0.640 | F _3,41_ = 0.32  P = 0.803 | F _3,41_ = 0.25  P = 0.857 | F _3,37_ = 2.71  P = 0.058 | F _3,37_ = 1.87  P = 0.150 | F _3,37_ = 1.97  P = 0.134 |
| I × D | F _1,41_ = 0.01  P = 0.915 | F _1,41_ = 0.01  P = 0.888 | F _1,41_ = 0.08  P = 0.778 | F _1,38_ = 4.86  **P = 0.033** | F _1,38_ = 0.36  P = 0.546 | F _1,38_ = 5.86  **P = 0.020** |
| G × D | F _3,41_ = 1.87  P = 0.148 | F _3,41_ = 0.42  P = 0.734 | F _3,41_ = 2.23  P = 0.098 | F _3,37_ = 0.42  P = 0.733 | F _3,37_ = 0.74  P = 0.534 | F _3,37_ = 0.28  P = 0.835 |
| I × G × D | F _3,41_ = 0.08  P = 0.968 | F _3,41_ = 0.82  P = 0.489 | F _3,41_ = 0.20  P = 0.889 | F _3,37_ = 0.89  P = 0.454 | F _3,37_ = 1.82  P = 0.159 | F _3,37_ = 1.43  P = 0.248 |
| Block | λ^2^ _1_ = 8.03  **P = 0.004** | λ^2^ _1_ = 10.4  **P = 0.001** | λ^2^ _1_ = 1.4 ^e-14^  P = 1 | λ^2^ _1_ = 1.32  P = 0.250 | λ^2^ _1_ = 1.32  P = 0.250 | λ^2^ _1_ = 5.6 ^e-14^  P = 1 |

| Root | | | | | | |
| --- | --- | --- | --- | --- | --- | --- |
| Factor | **With WSH** | | | **Without WSH** | | |
|  | Ascomycota | Basidiomycota | Zygomycota | Ascomycota | Basidiomycota | Zygomycota |
| R^2^ | 0.62 | 0.44 | 0.61 | 0.32 | 0.17 | 0.23 |
| I | F _1,5_ = 2.40  P = 0.172 | F _1,5_ = 2.55  P = 0.161 | F _1,5_ = 8.03  **P = 0.029** | F _1,5_ = 4.07  P = 0.093 | F _1,5_ = 0.02  P = 0.872 | F _1,5_ = 4.56  P = 0.079 |
| G | F _3,41_ = 0.18  P = 0.907 | F _3,41_ = 2.04  P = 0.122 | F _3,41_ = 0.90  P = 0.448 | F _3,37_ = 0.44  P = 0.719 | F _3,37_ = 0.17  P = 0.911 | F _3,37_ = 0.42  P = 0.734 |
| D | F _1,41_ = 69.7  **P = 0.000** | F _1,41_ = 2.83  P = 0.099 | F _1,41_ = 52.0  **P = 0.000** | F _1,39_ = 6.7  **P = 0.013** | F _1,39_ = 2.07  P = 0.158 | F _1,39_ = 3.15  P = 0.083 |
| I × G | F _3,41_ = 0.27  P = 0.842 | F _3,41_ = 1.36  P = 0.266 | F _3,41_ = 0.16  P = 0.916 | F _3,37_ = 0.16  P = 0.922 | F _3,37_ = 0.66  P = 0.577 | F _3,37_ = 0.02  P = 0.995 |
| I × D | F _1,41_ = 4.43  **P = 0.041** | F _1,41_ = 5.68  **P = 0.021** | F _1,41_ = 11.32  **P = 0.001** | F _1,40_ = 10.5  **P = 0.002** | F _1,40_ = 2.57  P = 0.116 | F _1,40_ = 5.33  **P = 0.026** |
| G × D | F _3,41_ = 0.90  P = 0.447 | F _3,41_ = 1.94  P = 0.136 | F _3,41_ = 2.00  P = 0.128 | F _3,37_ = 0.60  P = 0.614 | F _3,37_ = 0.75  P = 0.527 | F _3,37_ = 0.36  P = 0.781 |
| I × G × D | F _3,41_ = 2.22  P = 0.099 | F _3,41_ = 0.99  P = 0.406 | F _3,41_ = 0.73  P = 0.539 | F _3,37_ = 0.33  P = 0.8021 | F _3,37_ = 0.78  P = 0.508 | F _3,37_ = 0.34  P = 0.796 |
| Block | λ^2^ _1_ = 2.31  P = 0.127 | λ^2^ _1_ = 3.60  P = 0.057 | λ^2^ _1_ = 1.35  P = 0.243 | λ^2^ _1_ = 1.7 ^e-14^  P = 1 | λ^2^ _1_ = 0  P = 1 | λ^2^ _1_ = -1.6 ^e-14^  P = 1 |
|  |  |  |  |  |  |  |
| Leaf | | | | | | |
|  | **With WSH** | | | **Without WSH** | | |
|  | Ascomycota | Basidiomycota | Zygomycota | Ascomycota | Basidiomycota | Zygomycota |
| R^2^ | 0.47 | 0.28 | 0.55 | 0.67 | 0.46 | 0.65 |
| I | F _1,5_ = 3.07  P = 0.131 | F _1,5_ = 0.0  P = 0.767 | F _1,5_ = 3.74  P = 0.101 | F _1,4_ = 8.40  **P = 0.033** | F _1,5_ = 3.44  P = 0.116 | F _1,5_ = 0.00  P = 0.926 |
| G | F _3,37_ = 4.61  **P = 0.007** | F _3,37_ = 1.59  P = 0.205 | F _3,37_ = 4.08  **P = 0.013** | F _3,27_ = 1.00  P = 0.405 | F _3,26_ = 0.65  P = 0.585 | F _3,27_ = 1.49  P = 0.237 |
| D | F _1,38_ = 1.23  P = 0.273 | F _1,38_ = 11.0  **P = 0.001** | F _1,38_ = 1.82  P = 0.184 | F _1,27_ = 78.2  **P = 0.000** | F _1,26_ = 4.93  **P = 0.035** | F _1,27_ = 59.2  **P = 0.000** |
| I × G | F _3,37_ = 1.90  P = 0.144 | F _3,37_ = 0.04  P = 0.987 | F _3,37_ = 3.59  **P = 0.022** | F _3,28_ = 0.02  P = 0.993 | F _3,27_ = 0.36  P = 0.775 | F _3,27_ = 0.49  P = 0.686 |
| I × D | F _1,38_ = 1.51  P = 0.226 | F _1,38_ = 0.03  P = 0.862 | F _1,38_ = 2.54  P = 0.118 | F _1,27_ = 0.96  P = 0.334 | F _1,26_ = 0.32  P = 0.574 | F _1,27_ = 0.67  P = 0.416 |
| G × D | F _3,37_ = 1.97  P = 0.134 | F _3,37_ = 0.20  P = 0.893 | F _3,37_ = 3.78  **P = 0.018** | F _3,29_ = 1.28  P = 0.296 | F _3,27_ = 1.05  P = 0.384 | F _3,28_ = 4.04  **P = 0.016** |
| I × G × D | F _3,37_ = 3.29  **P = 0.030** | F _3,37_ = 1.98  P = 0.132 | F _3,37_ = 1.84  P = 0.155 | F _3,29_ = 1.903  P = 0.150 | F _3,27_ = 1.26  P = 0.306 | F _3,28_ = 0.72  P = 0.544 |
| Block | λ^2^ _1_ = 0.54  P = 0.459 | λ^2^ _1_ = 0  P = 1 | λ^2^ _1_ = 1.97  P = 0.160 | λ^2^ _1_ = 2.8 ^e-14^  P = 1 | λ^2^ _1_ = 3.85  **P = 0.049** | λ^2^ _1_ = 0.61  P = 0.432 |

| Seed Epiphytes | | | | | | |
| --- | --- | --- | --- | --- | --- | --- |
| Factor | **With WSH** | | | **Without WSH** | | |
|  | Ascomycota | Basidiomycota | Zygomycota | Ascomycota | Basidiomycota | Zygomycota |
| R^2^ | 0.25 | 0.25 | 0.18 | 0.39 | 0.39 | 0.18 |
| I | F _1,6_ = 1.47  P = 0.269 | F _1,6_ = 1.51  P = 0.264 | F _1,6_ = 0.95  P = 0.366 | F _1,5_ = 2.90  P = 0.138 | F _1,5_ = 2.92  P = 0.138 | F _1,5_ = 0.95  P = 0.366 |
| G | F _3,18_ = 2.47  P = 0.094 | F _3,18_ = 2.47  P = 0.094 | F _3,18_ = 0.88  P = 0.467 | F _3,18_ = 0.94  P = 0.439 | F _3,18_ = 0.94  P = 0.440 | F _3,18_ = 0.88  P = 0.467 |
| D | - | - | - | - | - | - |
| I × G | F _3,18_ = 0.49  P = 0.692 | F _3,18_ = 0.49  P = 0.690 | F _3,18_ = 0.01  P = 0.409 | F _3,18_ = 2.92  P = 0.062 | F _3,18_ = 2.92  P = 0.061 | F _3,18_ = 1.01  P = 0.409 |
| I × D | - | - | - | - | - | - |
| G × D | - | - | - | - | - | - |
| I × G × D | - | - | - | - | - | - |
| Block | λ^2^ _1_ = -4.2 ^e-14^  P = 1 | λ^2^ _1_ = -2.8 ^e-14^  P = 1 | λ^2^ _1_ = 0  P = 1 | λ^2^ _1_ = 0.39  P = 0.528 | λ^2^ _1_ = 0.36  P = 0.529 | λ^2^ _1_ = 0  P = 1 |
|  |  |  |  |  |  |  |
| Seed Endophytes | | | | | | |
|  | **With WSH** | | | **Without WSH** | | |
|  | Ascomycota | Basidiomycota | Zygomycota | Ascomycota | Basidiomycota | Zygomycota |
| R^2^ | 0.23 | 0.23 | 0.23 | 0.25 | 0.23 | 0.23 |
| I | F _1,4_ = 1.16  P = 0.340 | F _1,4_ = 1.17  P = 0.336 | F _1,4_ = 0.01  P = 0.952 | F _1,5_ = 0.21  P = 0.663 | F _1,5_ = 0.61  P = 0.466 | F _1,5_ = 0.61  P = 0.466 |
| G | F _3,11_ = 0.82  P = 0.507 | F _3,11_ = 0.82  P = 0.505 | F _3,11_ = 0.89  P = 0.472 | F _3,15_ = 2.46  P = 0.099 | F _3,15_ = 0.93  P = 0.448 | F _3,15_ = 0.93  P = 0.448 |
| D | - | - | - | - | - | - |
| I × G | F _3,11_ = 0.91  P = 0.466 | F _3,11_ = 0.91  P = 0.462 | F _3,11_ = 0.26  P = 0.850 | F _3,16_ = 0.27  P = 0.844 | F _3,16_ = 1.59  P = 0.229 | F _3,16_ = 1.59  P = 0.229 |
| I × D | - | - | - | - | - | - |
| G × D | - | - | - | - | - | - |
| I × G × D | - | - | - | - | - | - |
| Block | λ^2^ _1_ = 0  P = 1 | λ^2^ _1_ = -1.4 ^e-14^  P = 1 | λ^2^ _1_ = -2.8 ^e-14^  P = 1 | λ^2^ _1_ = 0.03  P = 0.852 | λ^2^ _1_ = 0  P = 1 | λ^2^ _1_ = 0  P = 1 |

**Figure S1.** Effect of the irrigation treatment (irrigated and non-irrigated) on yield (A) and seed Kernel weight (B) and protein contents (C) for four wheat genotypes grown in two agricultural fields with contrasting soil history: without water stress history (without WSH) and with water stress history (with WSH). Wheat genotype includes: AC Nass (*Triticum aestivum*), AC Walton (*Triticum aestivum*), AC Barrie (*Triticum aestivum*), Strongfield (*Triticum turgidum* subsp. durum). ANOVA tests for the effects of irrigation treatment, soil history, genotype and their interactions on yield, seed Kernel weight and protein contents are presented in Table 1.

**Figure S2.** The effects of developmental stages (for bulk soil, rhizosphere, leaf, root) and genotype (seed epiphytes) on bacteria Shannon diversity.


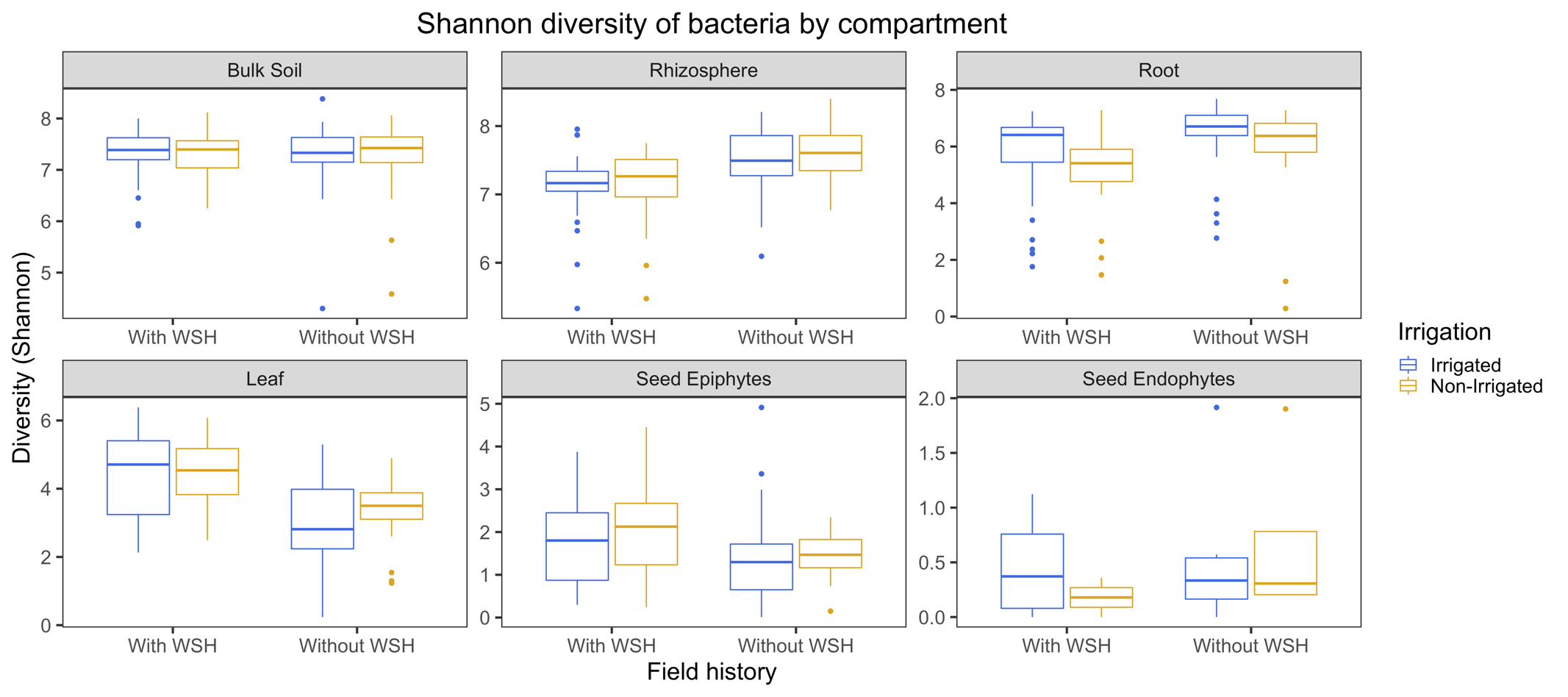


**Figure S3.** The effects of field history and irrigation treatment on bacteria Shannon diversity.

**Figure S4.** The effects of developmental stages (for bulk soil, rhizosphere, leaf, root) and genotype (seed epiphytes) on fungal Shannon diversity.


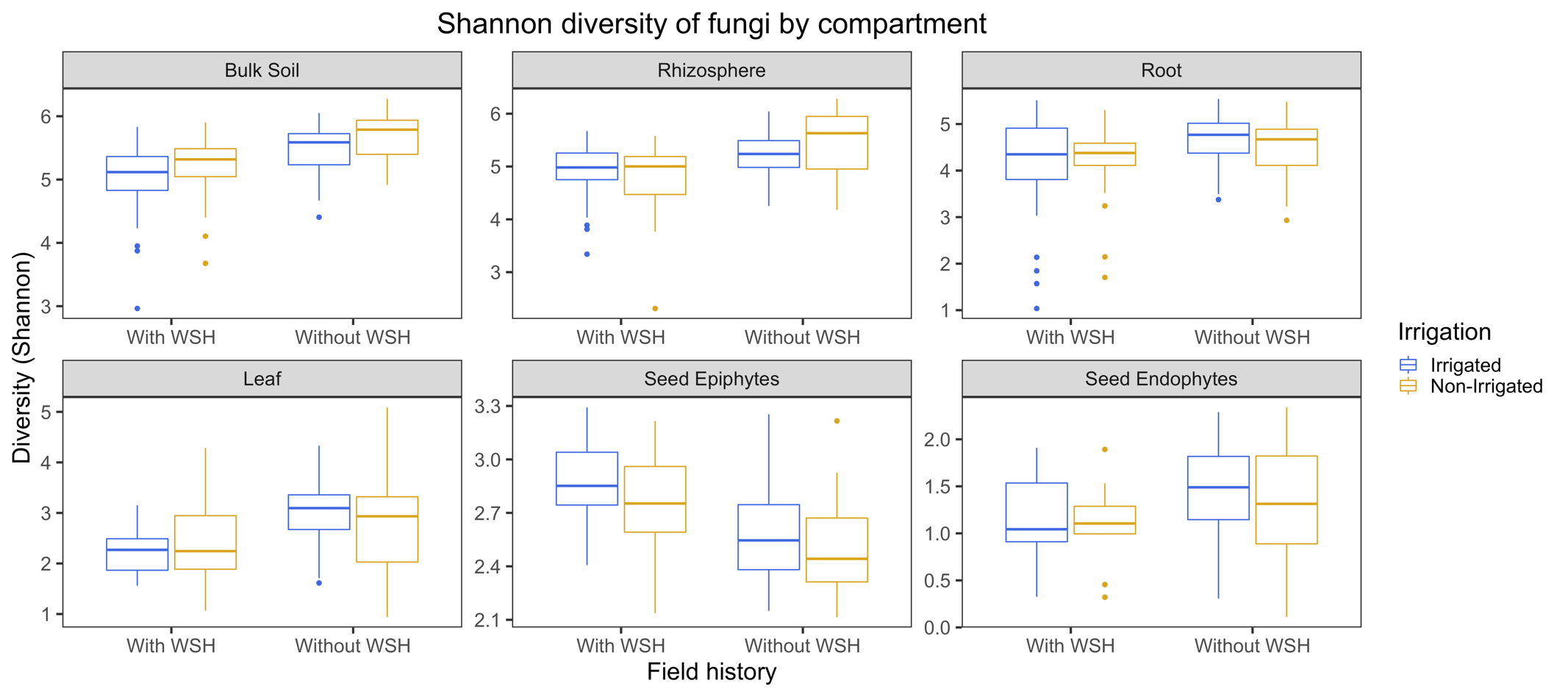


**Figure S5.** The effects of field history and irrigation treatment on fungi Shannon diversity.

**Figure S6.** Principal coordinate analyses (PCoA) of Bray–Curtis dissimilarity for bacterial (A) and fungal (B) communities.

**Figure S7.** Principal coordinate analyses (PCoA) of Bray–Curtis dissimilarity for bacterial.

**Figure S8.** Principal coordinate analyses (PCoA) of Bray–Curtis dissimilarity for bacterial.

**Figure S9.** Principal coordinate analyses (PCoA) of Bray–Curtis dissimilarity for fungal.

**Figure S10.** Principal coordinate analyses (PCoA) of Bray–Curtis dissimilarity for fungal.

**Figure S11.** Relative abundance of the most abundant bacterial phyla at order level associated with the bulk soil, rhizosphere, leaf, root and seed epiphytes of four wheat genotypes grown in fields without water stress history (without WSH) and with water stress history (with WSH) exposed to irrigation treatment (irrigated and non-irrigated). Developmental stages refer to post emergence, early stem elongation and early dough. Values represent the average of four replicates.

**Figure S12**. Relative abundance of the most abundant fungi phyla associated with the bulk soil, rhizosphere, leaf, root, seed epiphytes and endophytes of four wheat genotypes grown in fields without water stress history (without WSH) and with water stress history (with WSH) exposed to irrigation treatment (irrigated and non-irrigated). Developmental stages refer to post emergence, early stem elongation and early dough. Values represent the average of four replicates.

**Figure S13.** Relative abundance of the most abundant fungi at order level associated with the bulk soil, rhizosphere, leaf, root, seed epiphytes and endophytes of four wheat genotypes grown in fields without water stress history (without WSH) and with water stress history (with WSH) exposed to irrigation treatment (irrigated and non-irrigated). Developmental stages refer to post emergence, early stem elongation and early dough. Values represent the average of four replicates.
